# Exclusive detection of volatile aromatic hydrocarbons using bilayer oxide chemiresistors with catalytic overlayers

**DOI:** 10.1038/s41467-023-35916-3

**Published:** 2023-01-25

**Authors:** Seong-Yong Jeong, Young Kook Moon, Joseph Wang, Jong-Heun Lee

**Affiliations:** 1 Department of Materials Science and Engineering, Korea University, Seoul, 02841 Republic of Korea; 2 Department of Nanoengineering, University of California, San Diego, La Jolla, CA 92093 USA

**Keywords:** Sensors and biosensors, Electronic properties and materials, Synthesis and processing

## Abstract

The accurate detection and identification of volatile aromatic hydrocarbons, which are highly toxic pollutants, are essential for assessing indoor and outdoor air qualities and protecting humans from their sources. However, real-time and on-site monitoring of aromatic hydrocarbons has been limited by insufficient sensor selectivity. Addressing the issue, bilayer oxide chemiresistors are developed using Rh–SnO_2_ gas-sensing films and catalytic CeO_2_ overlayers for rapidly and cost-effectively detecting traces of aromatic hydrocarbons in a highly discriminative and quantitative manner, even in gas mixtures. The sensing mechanism underlying the exceptional performance of bilayer sensor is systematically elucidated in relation to oxidative filtering of interferants by the CeO_2_ overlayer. Moreover, CeO_2_-induced selective detection is validated using SnO_2_, Pt–SnO_2_, Au–SnO_2_, In_2_O_3_, Rh–In_2_O_3_, Au–In_2_O_3_, WO_3_, and ZnO sensors. Furthermore, sensor arrays are employed to enable pattern recognition capable of discriminating between aromatic gases and non-aromatic interferants and quantifying volatile aromatic hydrocarbon classifications.

## Abstract (web summary)

Bilayer sensors and sensor arrays with a catalytic CeO2 filter are proposed as a facile platform for high-performance gas sensors and electronic noses. Authors show the bilayer sensors enhance selectivity toward aromatic compounds, and the arrays provide comprehensive information such as gas concentration and composition.

## Introduction

**Fig. 1:**
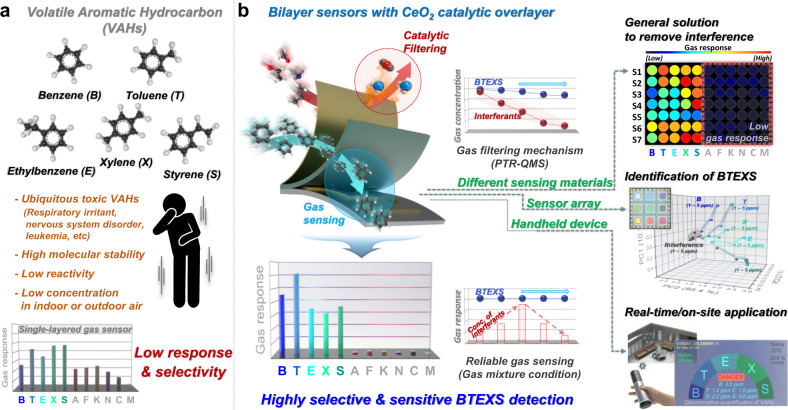
Concept of this study. **a** Obstacles for detecting volatile aromatic hydrocarbons (VAHs, i.e., benzene (B), toluene (T), ethylbenzene (E), xylene (X), and styrene (S)). **b** Bilayer sensor with catalytic CeO_2_ overlayer suggested as a universal solution for the exclusive detection of VAHs (left) and its potential for various applications (right).

With rapid societal and environmental changes, monitoring and managing new synthetic odors and various airborne chemicals have become increasingly important^1–6^. Volatile aromatic hydrocarbons (VAHs) such as benzene (B), toluene (T), ethylbenzene (E), xylene (X), and styrene (S) are major hazardous indoor and outdoor air pollutants in the petroleum and chemical industries, vehicles, and urban and residential areas (Fig. 1a)^7–10^. For instance, exposure to trace concentrations of benzene can cause leukemia, and methylbenzene (i.e., toluene and xylene), ethylbenzene, and styrene are representative health hazards that induce headaches, dizziness, nausea, and vomiting^7,8,10–12^. Furthermore, such hazardous VAHs are generally found at low concentrations (i.e., several ppm) in both indoor and outdoor environments^8,10,13^. Thus, the development of highly sensitive and selective techniques for detecting traces of VAHs must be developed for evaluating air quality in real-time and on-site.

Conventional VAH detection relies on bulky, expensive, complex, and time-consuming methods and instruments including gas chromatography–mass spectrometry (GC–MS)^14^, proton transfer reaction–mass spectrometry (PTR–MS)^15^, fluorescent probes^16^, and photoacoustic spectrometers^17^, which hamper instantaneous, portable, and cost-effective gas monitoring. In this regard, various chemiresistors including oxide semiconductors^1,18–26^, carbon nanotubes (CNTs)^27^, graphene-based materials (rGOs)^28^, transition metal dichalcogenides (TMDs)^29^, and transition metal carbides and nitrides (MXenes)^30^ have attracted considerable attention as alternative platforms due to their high gas response, rapid response, simple structure, good stability, and easy miniaturization. Among them, oxide semiconductor chemiresistors with higher operation temperatures are advantageous for VAHs detection because sufficient thermal activation can facilitate a sensing reaction between target gas and surface oxygen to induce charge transfer. However, oxide chemiresistive gas sensors often suffer from selective gas detection because the simple gas-sensing mechanism is based on charge transfer between the analyte gas and sensing surface, which becomes more crucial when attempting to detect stable gases such as VAHs. Indeed, most oxide chemiresistors such as SnO_2_, Co_3_O_4_, ZnO, TiO_2_, and In_2_O_3_ exhibit lower gas responses to VAHs than to highly reactive interfering gases such as ethanol, formaldehyde (HCHO), and acetone^31–34^ and often cannot identify or quantify specific chemical species in complex gas mixtures. This problem is related to the molecular stability of aromatic compounds comprising conjugated planar ring systems wherein electrons are strongly delocalized around the rings^35,36^. To overcome this limitation, although many approaches such as catalyst addition^37^, nanostructure adoption^31,38^, heterocomposite implementation^39^, electronic nose utilization^40^, and preconditioning part application^41^ have been explored, their capabilities for discriminating and quantifying aromatic BTEXS remains insufficient for monitoring air quality.

In light of this, assembling catalytic filtering layers (such as Co_3_O_4_^42^, TiO_2_^43^, SnO_2_^43^, Rh/TiO_2_^44^, Au^45^, Pt-loaded Al_2_O_3_^46^, and an external WO_3_ packed bed^47^) with gas-sensing films has recently spotlighted as a viable and facile solution for achieving gas selectivity toward aromatic gases by oxidation of the analyte prior to the gas-sensing reaction. Sensors fabricated with catalytic filtering layers are very advantageous because they exhibit highly tunable gas selectivities and responses by independently controlling sensing and catalytic reactions. However, designing sensors with catalytic filtering layers is still in the nascent stage, and high gas-sensing response and selectivity have never been simultaneously achieved toward aromatic BTEXS gases. In addition, sensors should be integrated into portable devices for monitoring air quality on-site and in real-time, which is difficult utilizing external filters such as those used in some previous studies.

Herein, we report a rational and facile strategy for ultraselectively and ultrasensitively detecting traces of aromatic BTEXS gases utilizing a bilayer sensor designed with a catalytic filtering layer (Fig. 1b). The key concept of the bilayer sensor is the catalytic oxidation of highly reactive nonaromatic interferants to less- or non-reactive species prior to the gas-sensing reaction. Owing to its moderate catalytic activity, CeO_2_ was employed as the overlayer material. The main advantage of the bilayer sensor over conventional gas detectors is that cross-responses to highly reactive interfering gases are removed while maintaining high gas responses to aromatic compounds, thereby enabling traces of aromatic BTEXS gases to be discriminated both selectively and quantitatively, even in gas mixtures. The mechanism underlying the exceptional sensing selectivity and the response was systematically studied for the sensing materials, catalytic overlayer, and bilayer structural configuration and validated by monitoring the conversion of analyte gases using a proton transfer reaction–quadrupole mass spectrometer (PTR–QMS). Moreover, the CeO_2_-coated bilayer sensor design was validated by enhancing the BTEXS-sensing properties of diverse gas sensors using SnO_2_, Pt–SnO_2_, Au–SnO_2_, In_2_O_3_, Rh–In_2_O_3_, Au–In_2_O_3_, WO_3_, and ZnO. In addition, we demonstrated that a bilayer sensor array with a catalytic CeO_2_ overlayer exhibited high or partial selectivity to aromatic compounds but a low gas response to other indoor pollutants to clearly identify BTEXS gases in a highly quantitative and discriminative manner. This study will open various pathways for developing highly precise, reliable, and cost-effective sensors to protect humans from the harmful effects of volatile aromatic compounds.

## Results

### Fabrication and characterization of gas-sensing films with a catalytic overlayer

**Fig. 2:**
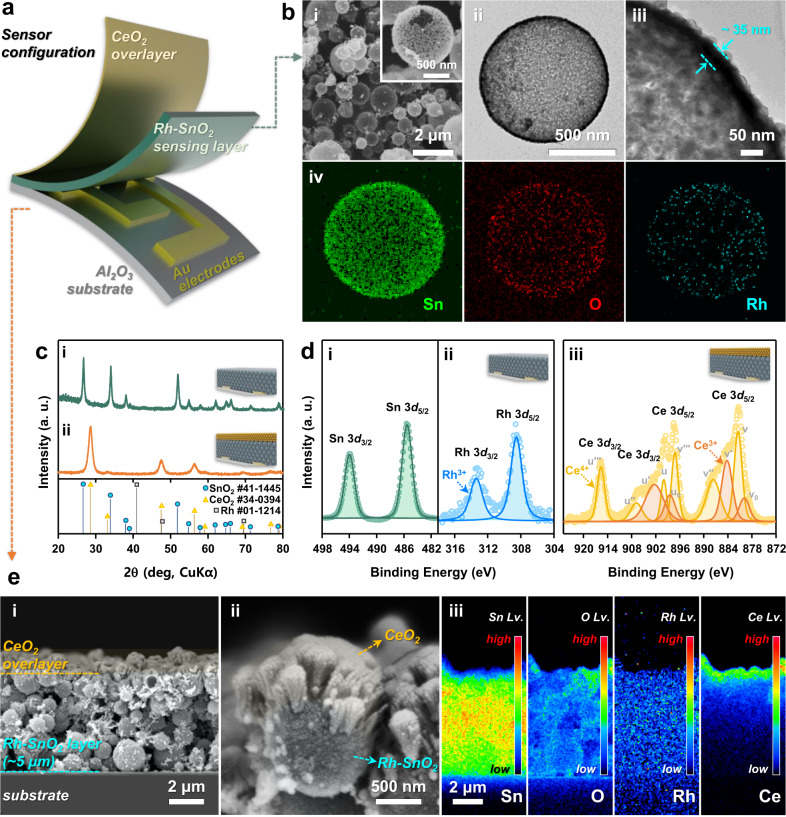
Morphology and phase characterizations of Rh–SnO_2_ and CeO_2_/Rh–SnO_2_ sensors. **a** Schematic of CeO_2_/Rh–SnO_2_ bilayer sensor. **b** Characterization of CeO_2_/Rh–SnO_2_ bilayer sensor (i) SEM, (ii and iii) TEM, and (iv) EDS elemental (Sn, O, and Rh) mapping images of Rh–SnO_2_ powders. **c** XRD patterns of (i) Rh–SnO_2_ and (ii) CeO_2_/Rh–SnO_2_ films. **d** XPS spectrums of the samples. (i) Sn 3*d* and (ii) Rh 3*d* XPS spectra for Rh–SnO_2_ film; (ii) Ce 3*d* XPS spectrum for CeO_2_/Rh–SnO_2_ film. **e** (i) Cross-sectional SEM, (ii) high-magnification SEM, and (iii) electron-probe microanalysis (EPMA) elemental mapping (Sn, O, Rh, and Ce) images of CeO_2_/Rh–SnO_2_ film. Source data are provided as a Source Data file.

A schematic of the monolithic bilayer sensor comprising an Rh–SnO_2_ sensing film and catalytic CeO_2_ overlayer is illustrated in Fig. 2a. The Rh–SnO_2_ sensing layer was screen-printed onto an Al_2_O_3_ substrate (1.5 mm × 1.5 mm) with two Au sensing electrodes (electrode gap: 0.2-mm gap), and the CeO_2_ overlayer was coated by electron-beam (e-beam) evaporation. The Rh–SnO_2_ hollow spheres were synthesized by ultrasonic spray pyrolysis and subsequent annealing at 600 °C. Detailed information regarding the sensor fabrication is described in the “Methods” section and Supplementary Note 1. The mean diameter of the ~80 Rh–SnO_2_ spheres was 0.84 ± 0.37 μm (Fig. 2b(i)). The broken part of the sphere exhibited a hollow morphology (Fig. 2b(i) inset), which was further confirmed by the bright and dark contours at the central part and outer shell (~35 nm thick) of the spheres, respectively, in the transmission electron microscopy (TEM) images (Fig. 2b(ii), b(iii)). The Brunauer–Emmett–Teller (BET) specific surface area of the Rh–SnO_2_ hollow spheres was 35.6 m^2^ g^–1^, and the N_2_ adsorption and desorption isotherms exhibited type IV characteristics with H3 hysteresis loops (Supplementary Fig. 1). These results indicate that the hollow Rh–SnO_2_ spherical structure is highly accessible to gases. The tetragonal SnO_2_ phase was confirmed by the high-resolution TEM (HR-TEM) image exhibiting lattice patterns with interplanar spacings of 3.31 and 2.69 Å corresponding to the (110) and (101) planes (Supplementary Fig. 2), respectively, and by the corresponding X-ray diffraction (XRD) patterns (Fig. 2c(i)). No Rh-related phase appeared in the HR-TEM image, nor was indicated by the corresponding XRD pattern probably due to the low detection limit of X-ray diffractometer. However, the energy-dispersive spectroscopy (EDS) elemental mappings revealed that Rh, Sn, and O were uniformly distributed over the entire particle surface (Fig. 2b(iv)). Moreover, a peak corresponding to Rh^3+^ appeared in the X-ray photoelectron spectroscopy (XPS) profile, confirming that Rh was present as Rh_2_O_3_ (Fig. 2d(ii))^48,49^.

An ~0.4-μm-thick CeO_2_ overlayer was coated on the ~5-μm-thick Rh–SnO_2_ gas-sensing film (Fig. 2e(i)), as estimated from the SEM image of the film deposited on the planar SiO_2_/Si wafer (Supplementary Fig. 3). A high-magnification cross-sectional scanning electron microscopy (SEM) image of the bilayer film uppermost region further shows that the Rh–SnO_2_ powder spheres were covered with CeO_2_ nanoparticles (Fig. 2e(ii)). Electron-probe microanalysis (EPMA) elemental mapping (Fig. 2e(iii)) revealed that Sn, O, and Rh were distributed throughout the entire film, whereas Ce was only distributed in the top region. The Rh–SnO_2_ film with the CeO_2_ overlayer was further analyzed using XRD and XPS (Fig. 2c(ii), d(iii) and Supplementary Fig. 4). The CeO_2_ overlayer was identified as a cubic fluorite phase (ICDD #34-0394). The Rh–SnO_2_-related peaks disappeared in the XRD pattern and XPS spectrum of the CeO_2_-overlayer-coated Rh–SnO_2_ film because of the low detection depth (i.e., few-nanometer depth resolution) of both XRD and XPS (Fig. 2c(ii) and Supplementary Fig. 4).

### Exclusive detection of volatile aromatic hydrocarbons using bilayer sensors with a porous Rh–SnO_2_ sensing film and a CeO_2_ overlayer

**Fig. 3:**
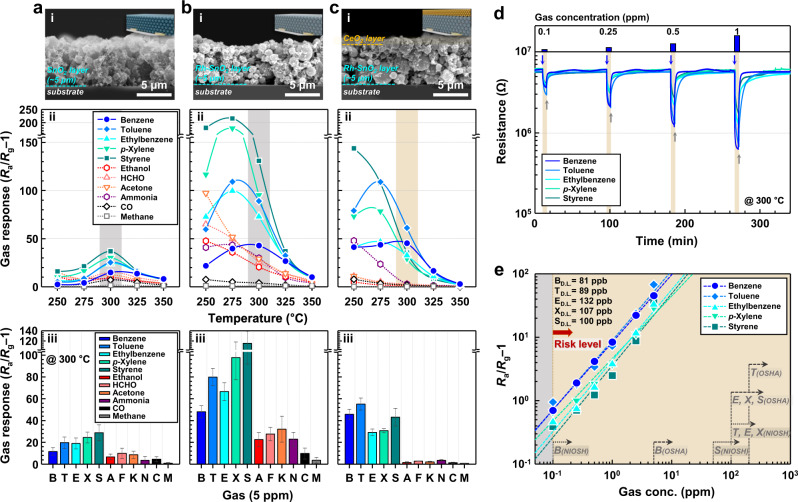
Gas-sensing performance of the samples. **a**–**c** Cross-sectional SEM images and gas-sensing characteristics of SnO_2_ (**a**), Rh–SnO_2_ (**b**), and 0.4CeO_2_/Rh–SnO_2_ (**c**) sensors (analyte gas concentration: 5 ppm; error bars indicate standard deviations for three sensors). **d** Dynamic gas-sensing transients of 0.4CeO_2_/Rh–SnO_2_ sensor exposed to 0.1–1 ppm BTEXS at 300 °C. **e** Gas responses as functions of BTEXS concentration and exposure limits of volatile aromatic compounds based on acute and chronic inhalation or working conditions (The dashed lines are fitted curves; the linear regression equations are expressed as y = 0.859× + 1.017 (B), y = 0.917× + 1.045 (T), y = 0.816x + 0.797 (E), y = 0.750× + 0.806 (X), y = 0.809× + 0.745 (S), where y is the gas response and x is the gas concentration). Source data are provided as a Source Data file.

The gas-sensing characteristics of the CeO_2_/Rh–SnO_2_ bilayer film were measured in the range 250–350 °C for detecting 5 ppm of VAHs (B, T, E, X, and S) and nonaromatic interference gases (ethanol [A], formaldehyde [F], acetone [K], ammonia [N], carbon monoxide [C], and methane [M]) (Fig. 3c). To elucidate the roles of the Rh loading and CeO_2_ overlayer, pure ~5-μm-thick SnO_2_ and Rh–SnO_2_ films, which were similar thickness to that of the Rh–SnO_2_ gas-sensing film in the bilayer sensor, were also prepared (Fig. 3a(i), b(i) and Supplementary Fig. 5), and their gas-sensing characteristics were investigated (Fig. 3a(ii), (iii) and b(ii), (iii)). All three sensors exhibited *n*-type oxide semiconductor behavior with decreased resistance in a reducing atmosphere (Supplementary Fig. 6). Thus, the gas response (*S*) was defined by Eq. (1) as follows:

1 $$({R}_{{{{{{\rm{a}}}}}}}-{R}_{{{{{{\rm{g}}}}}}})/{R}_{{{{{{\rm{g}}}}}}}={R}_{{{{{{\rm{a}}}}}}}/{R}_{{{{{{\rm{g}}}}}}}-1$$

where *R*_a_ and *R*_g_ are the resistances in the air and analyte gas, respectively. See Supplementary Note 1 for the detailed gas-sensing measurements.

The responses to 5 ppm of the various analyte gases are plotted as functions of sensing temperature for the pure SnO_2_ sensor (Fig. 3a). Clearly, all the response curves are bell-shaped, and the pure SnO_2_ sensor exhibited the highest VAH responses (*S*_B_, *S*_T_, *S*_E_, *S*_X_, and *S*_S_ = 14.9, 25.3, 24.3, 30.2, and 36.9, respectively) at 300 °C. The aromatic gas responses of SnO_2_ sensor were comparable to nonaromatic interfering gases, thereby hindering selective VAH detection. These results are consistent with the typical sensing characteristics of pure SnO_2_ reported in the literature^31,38,50,51^. The Rh–SnO_2_ sensor exhibited considerably enhanced gas responses to most of the analyte gases at all the sensing temperatures (Fig. 3b). For example, at 300 °C, the gas responses of the Rh–SnO_2_ sensor to 5 ppm of the volatile aromatic hydrocarbons (B, T, E, X, and S) increased to 42.8, 89.1, 73.0, 95.2, and 130.9, respectively, which were ~2.9–3.5-fold higher than those of the pure SnO_2_ sensor (Fig. 3b(iii)). The Rh loading-induced increased overall gas response can be explained by the decreased background charge-carrier concentrations caused by the *p*–*n* junction and/or Schottky contact formed between the *n*-type SnO_2_ and *p*-type Rh_2_O_3_, which work functions are 4.9 and 5.0 eV, respectively^52–54^, and is supported by the Rh loading increasing the sensor resistance from 0.9 to 2.6 MΩ in the air at 300 °C (Supplementary Fig. 6b). In addition, the chemisorbed oxygen species (O^–^_ads_)/lattice oxygen species (O^–^_lat_) peak ratio for the Rh–SnO_2_ sensor was substantially higher than that for the pure SnO_2_ sensor (Supplementary Fig. 7), indicating that Rh-induced oxygen spillover was also responsible for the increased response. Notably, the gas responses of the Rh–SnO_2_ sensor were considerably higher than the Pd–SnO_2_, Pt–SnO_2_, and Au–SnO_2_ sensor counterparts (Supplementary Note 2 and Supplementary Figs. 8, 9), indicating that among the noble-metal catalysts, Rh was the most effective for promoting VAH sensing. However, because loading of Rh to SnO_2_ enhanced responses to not only VAHs but also nonaromatic interference gases (Fig. 3b), the Rh–SnO_2_ sensor was still insufficient for selectively detecting VAHs.

In contrast, coating a 0.4-μm-thick CeO_2_ overlayer on the Rh–SnO_2_ film surface (hereafter, 0.4CeO_2_/Rh–SnO_2_) considerably decreased the interfering-gas responses, whereas the VAH responses remained high (Fig. 3c). It is worth noting that the 0.4CeO_2_/Rh–SnO_2_ sensor selectively and sensitively detected VAHs over a wide sensing-temperature range (275–350 °C). In particular, the sensor exhibited remarkably high gas responses of *S*_B_, *S*_T_, *S*_E_, *S*_X_, and *S*_S_ = 45.3, 61.2, 32.7, 29.2, and 35.4, respectively, to 5 ppm VAHs and negligible interfering-gas cross-responses of *S*_A_, *S*_F_, *S*_K_, *S*_N_, *S*_C_, and *S*_M_ = 1.0, 3.0, 2.1, 2.9, 1.9, and 0.3, respectively, at 300 °C, thereby demonstrating exclusive aromatic hydrocarbon detection. To investigate the catalytic CeO_2_ overlayer thickness effect on VAH detection, Rh–SnO_2_ sensors were fabricated with different CeO_2_ overlayer thicknesses: 0.05CeO_2_/Rh–SnO_2_, 0.1CeO_2_/Rh–SnO_2_, and 0.7CeO_2_/Rh–SnO_2_ (Supplementary Figs. 10,11). All the sensors selectively and sensitively detected VAHs over a wide temperature range (Supplementary Figs. 11,12 and Supplementary Note 3), clearly indicating that coating the gas-sensing film with a CeO_2_ overlayer is a promising strategy for exclusively detecting VAHs. At 300 °C, because the 0.4CeO_2_/Rh–SnO_2_ sensor exhibited the highest VAH selectivity (e.g., VAH selectivity increased with increasing CeO_2_ coating thickness to 0.4 μm and subsequently decreased with further increasing CeO_2_ coating thickness from 0.4 to 0.7 μm) (Supplementary Fig. 12), the optimal CeO_2_ overlayer thickness and operating temperature of the bilayer sensor were fixed at 0.4 μm and 300 °C, respectively. In addition, a similar tendency in gas responses was further achieved from each sensor batch (Supplementary Fig. 13), which confirms the excellent sensor reproducibility.

To demonstrate the potential of 0.4CeO_2_/Rh–SnO_2_ as an indoor air-monitoring sensor, the cyclic sensing characteristics to 0.1–1 ppm of aromatic hydrocarbons (BTEXS) were examined at 300 °C (Fig. 3d). Moreover, from the graph of the gas response plotted as a function of the gas concentration, the detection limit (DLs) of aromatic hydrocarbons were calculated using the sensing criterion *R*_a_/*R*_g_ – 1 > 0.2 as 81 ppb (B), 89 ppb (T), 132 ppb (E), 107 ppb (X), and 100 ppb (S), respectively (Fig. 3e)^55,56^. The low detection limits of 0.4CeO_2_/Rh–SnO_2_ sensor were further estimated to be 16 ppb (B), 62 ppb (T), 114 ppb (E), 75 ppb (X), and 52 ppb (S) when 3*(*RMS*_noise_/Slope) was used as the criterion for gas sensing^57^ or 0.017 ppb (B), 0.019 ppb (T), 0.020 ppb (E), 0.014 ppb (X), and 0.037 ppb (B) when a signal-to-noise ratio >5 was used as the sensing criterion^30,58^, respectively (Fig. 3e). All the theoretical detection limits were considerably lower than the permissible exposure limits for the 8 h time-weighted average (TWA) established by the National Institute for Occupational Safety and Health (NIOSH) and Occupational Safety and Health Administration (OSHA)^59–63^, indicating that the 0.4CeO_2_/Rh–SnO_2_ sensor can be used to accurately monitor sub-ppb levels of hazardous aromatic hydrocarbons. Furthermore, the stability of the 0.4CeO_2_/Rh–SnO_2_ bilayer sensor was verified by reproducing sensing properties during seven consecutive cycles and long-term cycling for 24 days (Supplementary Fig. 14).

The ambient humidity effect of the 0.4CeO_2_/Rh–SnO_2_ sensor, which is important for their practical applications such as indoor air quality monitoring, was also investigated by measuring gas sensing characteristics under simulated indoor conditions (static atmosphere; relative humidity (RH) 50%; temperature 18 °C) (Supplementary Fig. 15). For this, the relative humidity of an acrylic chamber (inner volume: 50 cm × 50 cm × 50 cm) was controlled by a humidifier and the analyte gases were filled into the chamber by evaporating the high-purity solutions (benzene, toluene, ethylbenzene, *p*-xylene, styrene, and ethanol). The final concentrations of analytes were fixed at 5 ppm. The detailed measurement was conducted using the following procedures: when the sensor resistance was constant, the sensor was injected into the chamber and the variation of sensor resistance was measured for 100 s, subsequently recovered to its original resistance via ejecting the sensor from the chamber. The 0.4CeO_2_/Rh–SnO_2_ sensor exhibited high selectivity toward VAHs over ethanol (*S*_B_/*S*_A_ = 1.4, *S*_T_/*S*_A_ = 5.5, *S*_E_/*S*_A_ = 5.8, *S*_X_/*S*_A_ = 5.1, and *S*_S_/*S*_A_ = 3.5, respectively) under the simulated conditions (RH 50%) (Supplementary Fig. 15b). Although the decrease of sensor response was observed in the simulated atmosphere, the gas selectivity toward VAHs remained substantial. These results demonstrate that the fabricated sensor can be used to detect sub-ppm-level VAHs in a real environment.

### Exceptional high performance in gas mixtures

**Fig. 4:**
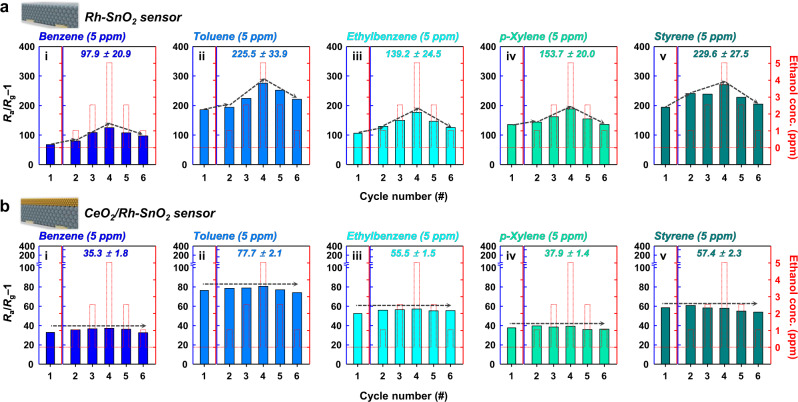
Multi-component gas-sensing analysis. **a**, **b** Gas responses of the Rh–SnO_2_ (**a**) and the 0.4CeO_2_/Rh–SnO_2_ (**b**) sensors to (i) gas mixture of 5 ppm benzene (filled bars) and 0–5 ppm ethanol (dashed red lines), (ii) gas mixture of 5 ppm toluene (filled bars) and 0–5 ppm ethanol (dashed red lines), (iii) gas mixture of 5 ppm ethylbenzene (filled bars) and 0–5 ppm ethanol (dashed red lines), (iv) gas mixture of 5 ppm *p*-xylene (filled bars) and 0–5 ppm ethanol (dashed red lines), and (v) gas mixture of 5 ppm styrene (filled bars) and 0–5 ppm ethanol (dashed red lines) at 300 °C. Note that the Rh-SnO_2_ sensor showed a concentration-dependent response variations toward ethanol, while the response of the 0.4CeO_2_/Rh–SnO_2_ sensor was barely affected by the coexistence of 0–5 ppm of ethanol. Source data are provided as a Source Data file.

Under actual gas-sensing conditions, volatile aromatic compounds (BTEXS) coexist with different concentrations of airborne chemicals, which often hinders quantifying gas concentrations^1,2,12,64^. To determine the reliable sensing under interferant, the sensor responses to 5 ppm of pure B, T, E, X, or S and the same in mixed gases were measured at 300 °C by varying the interfering-gas type and concentration i.e., 0.1–5 ppm of ethanol, HCHO, or acetone (Fig. 4, Supplementary Fig. 16, and Supplementary Note 4). For the Rh–SnO_2_ sensor, the gas responses to 5 ppm of B, T, E, X, or S varied considerably depending on the interfering-gas concentration in the gas mixture (i.e., the B, T, E, X, and S responses showed average variations of 21.3, 15.0, 17.6, 13.0, and 12.0%, respectively, as shown in Fig. 4a and Supplementary Fig. 16b, d). In stark contrast, the 0.4CeO_2_/Rh–SnO_2_ sensor exhibited nearly identical B, T, E, X, or S gas responses, regardless of the interfering gas (i.e., the B, T, E, X, and S responses showed average variations of 5.1, 2.7, 2.7, 3.7, and 4.0%, respectively, under the same experimental conditions, as shown in Fig. 4b and Supplementary 16c, e). This result confirms the exceptional selectivity for aromatic hydrocarbons in gas mixtures, which originates from the distinctive bilayer design with a catalytic CeO_2_ overlayer.

### Gas-sensing mechanism and catalytic characterization

**Fig. 5:**
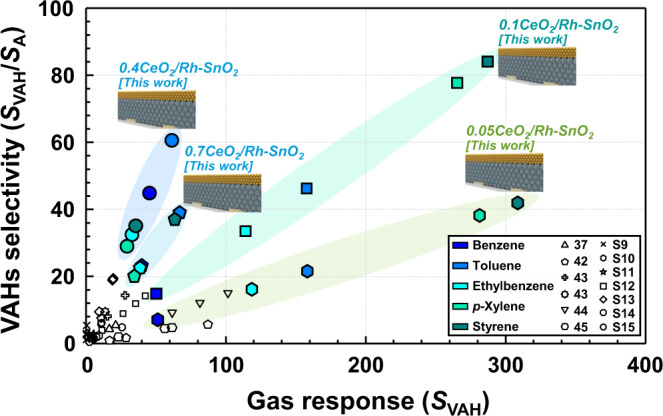
Gas-sensing performance of CeO_2_/Rh–SnO_2_ bilayer sensors. Selectivities (*S*_VAH_/*S*_ethanol_) and responses (*S*_B_, *S*_T_, *S*_E_, *S*_X_, and *S*_S_) to VAHs measured in this study compared to those previously reported in the literature^37,42–45^^,S9–S23^. Source data are provided as a Source Data file.

Notably, exclusively detecting aromatic hydrocarbons with negligible cross-responses to nonaromatic interference gases can hardly be achieved using common single-layered gas sensors (e.g., gas-sensing films comprising uniformly doped/loaded noble-metal or oxide catalysts and nanostructured building blocks)^31,37–39,52–54^. This is supported by the relatively low gas response and selectivity of the common single-layered sensors such as Au-loaded hierarchical MoO_3_ hollow spheres^37^, CuO nanoparticles^S9^, CuO/SnO_2_ composites^S10^, SnO_2_/V_2_O_5_ composites^S11^, Hexagonal WO_3_ nanosheets^S12^, Triangular-CeO_2_-nanoflakecoated ZnO sensor^S13^, Cr-doped Co_3_O_4_ nanorods^S14^, Pd-decorated SnO_2_ nanowires^S15^ (Fig. 5). However, both high gas responses (*S*_VAH_) and excellent selectivities (*S*_VAH_/*S*_A_) toward aromatic BTEXSs were simultaneously achieved using monolithic bilayer sensors with a catalytic CeO_2_ overlayer, and are the highest values among those reported in the literature (Fig. 5 and Supplementary Table 1)^37,42–45^^,S9–S23^. The intriguing gas-sensing characteristics of the bilayer sensor can be explained by gas filtering through the CeO_2_ catalytic overlayer because the gas-sensing layer (bottom) and catalytic overlayer (top) were completely separated. In the bilayer sensor structure, the analyte gases were transported to the bottom sensing electrodes through the top CeO_2_ overlayer, and the bottom Rh–SnO_2_ film was mainly responsible for the gas-sensing reaction. Thus, the top CeO_2_ layer transformed the analyte gases into less- or non-reactive forms through catalytic oxidation, which explains why the gas response decreased when the Rh–SnO_2_ film was coated with the CeO_2_ overlayer.

Accordingly, the gas responses to both VAHs and other representative interfering gases (e.g., ethanol, formaldehyde, and acetone) were plotted as functions of the CeO_2_ layer thickness (Supplementary Fig. 17). At all the sensing temperatures (250–350 °C), the gas responses to both the VAHs and interfering gases gradually decreased with increasing CeO_2_ overlayer thickness. Despite nearly the same Rh–SnO_2_-sensing-film thickness being employed, the decreased gas response suggests that the gases were oxidatively consumed at the CeO_2_ overlayer, which is plausible because CeO_2_ exhibits Ce^3+^ and Ce^4+^ multivalencies and abundant oxygen vacancies and, therefore, excellent thermocatalytic activity for oxidizing reducing gases^65^. Notably, Ce^4+^ interacts with C-, H-, N-, or O-atom-containing reducing gases and is reduced to Ce^3+^, thereby oxidizing the reducing gas to less- or non-reactive forms (e.g., CO_2_, H_2_O, or N_2_) through Reaction (2) as follows^66,67^:

2 $${{{{{{\rm{Ce}}}}}}}^{4+}+{{{{{\rm{C}}}}}}-,{{{{{\rm{H}}}}}}-,{{{{{\rm{N}}}}}}-,\, {{{{{\rm{or}}}}}}\,{{{{{\rm{O}}}}}}-{{{{{\rm{containing}}}}}}\,{{{{{\rm{reducing}}}}}}\,{{{{{\rm{gases}}}}}}\to {{{{{{\rm{Ce}}}}}}}^{3+}+{{{{{{\rm{CO}}}}}}}_{2}+{{{{{{\rm{H}}}}}}}_{2}{{{{{\rm{O}}}}}}+{{{{{{\rm{N}}}}}}}_{2}.$$

In addition, the CeO_2_ oxygen vacancies promote oxygen adsorption, which facilitates reducing gas oxidation through Reaction (3) as follows^66,67^:

3 $$[2{{{{{{\rm{Ce}}}}}}}^{3+},{{{{{{{\rm{V}}}}}}}_{{{{{{\rm{O}}}}}}}}^{\cdot \cdot }]+{{{{{{\rm{O}}}}}}}_{2}\to [{{{{{{\rm{Ce}}}}}}}^{4+},{{{{{{\rm{Ce}}}}}}}^{3+}]+{{{{{{\rm{O}}}}}}}^{2-}.$$

To determine whether multivalencies and oxygen vacancies, which might be affected by gas oxidation, formed in the CeO_2_, XPS, and electron paramagnetic resonance (EPR) analysis were carried out and shown in Fig. 2d(iii) and Supplementary Fig. 18, respectively. As demonstrated in the XPS measurement (Fig. 2d(iii)), the coexistence of Ce^3+^ and Ce^4+^ in CeO_2_ was confirmed by Ce 3*d* spectra observation. Furthermore, the EPR spectrum exhibited a peak at *g* ≈ 1.960, indicating that the CeO_2_ also exhibited oxygen vacancies (Supplementary Fig. 18)^68^.

However, these results cannot elucidate the remarkably high selectivity of the CeO_2_/Rh–SnO_2_ sensors toward aromatic compounds, and the chemiresistive variation induced by CeO_2_ depends on the gas species. Considering that aromatic compounds with benzene ring are known to be very stable whereas the non-volatile aromatic compounds gases are more reactive than VAHs^36^, the highly selective VAH detection can be associated with the complete oxidation of highly reactive nonaromatic gases and survival of less reactive BTEXS gases during transport across the CeO_2_ overlayer. This is supported by the fact that, the responses to aromatic BTEXS gases decreased slightly, whereas those to nonaromatic gases decreases down to negligible levels, as shown in Fig. 3b, c, respectively, thereby leading to selective and sensitive VAH detection.

The sensor temperature required to achieve the maximum gas response (*T*_M_) also supports the proposed mechanism. Oxide chemiresistor response curves plotted as functions of sensing temperature are usually bell-shaped^12,69,70^. For instance, in the low-sensing-temperature region, the gas response gradually increased through the thermal promotion of the sensing reaction between the analyte gas and ionized surface O atoms. At excessively high sensing temperatures, the gas response decreased because the analyte gases were oxidized to nonreactive species (e.g., CO_2_ or H_2_O) at the upper part of the gas-sensing film before diffusing to the bottom sensing region near the electrodes. Since the gas-sensing reactions of more-reactive gases (e.g., ethanol, HCHO, and acetone) can be promoted at lower temperatures, the higher gas reactivity reduces *T*_M_. Notably, the *T*_M_ values for benzene and the other aromatic TEXS gases were 300 and 275 °C, respectively, which are considerably higher than those of other nonaromatic gases (*T*_M_ ≤ 250 °C) (Fig. 3b(ii)). This indicates that the catalytic CeO_2_ layer can selectively remove highly reactive nonaromatic gases at moderate sensing temperatures (e.g., 300 and 275 °C) without expending VAHs because the oxidative consumption of highly reactive interfering gases begins at a relatively low temperature (≤250 °C) (Fig. 3b(iii)).

The effects of the CeO_2_ overlayer thickness on the gas sensing characteristics are also important for understanding the oxidative consumption of interfering gases. The single-layer Rh–SnO_2_ sensor exhibited high and comparable responses to a wide range of reducing gases. In contrast, all four sensors coated with 0.05–, 0.1–, 0.4–, and 0.7–μm–thick CeO_2_ overlayers showed high gas selectivity and response to VAHs, confirming the interfering gases were successfully filtered by catalytic oxidation in the CeO_2_ overlayer. For example, the thin (0.05– and 0.1–μm–thick) CeO_2_ overlayers led to relatively high cross-responses to interfering gases, probably due to insufficient oxidation of interfering gases, resulting in low selectivity to VAHs (Supplementary Figs. 12 and 13). However, excessive thickening of (0.7–μm–thick) CeO_2_ overlayer significantly reduced the gas responses to both VAHs and interfering gases (Supplementary Figs. 11c, f and 13f), and this could be related to the catalytic oxidation of all analyte gases, including the VAHs. All these explanations support that the CeO_2_ overlayer coatings are effective for oxidative filtering of interfering gases, but the careful control of the CeO_2_ overlayer thickness could be crucial for achieving high selectivity and response to VAHs.

**Fig. 6:**
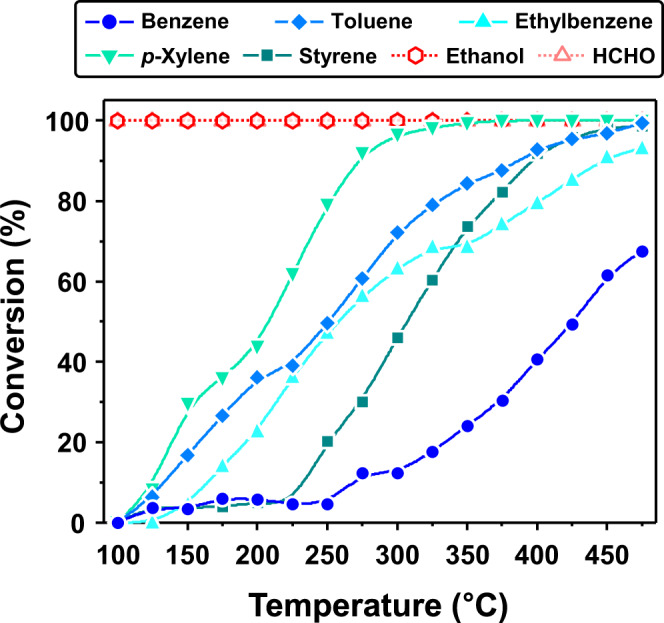
Catalytic performance of CeO_2_. Catalytic conversion of 5 ppm of different analyte gases over CeO_2_ catalyst as functions of temperature in the range 100–475 °C. Source data are provided as a Source Data file.

To elucidate the gas-sensing mechanism, the catalytic activity of CeO_2_ nanoparticles was investigated by converting (*η*) 1 ppm of benzene, toluene, ethylbenzene, *p*-xylene, and styrene as well as interfering ethanol and HCHO using a PTR–QMS (Fig. 6, Supplementary Note 5, and Supplementary Fig. 19). For this, 0.1 g of CeO_2_ nanoparticles were loaded onto quartz wool in the middle of a furnace. The interference gases (ethanol and HCHO) were completely removed below 100 °C, whereas the VAH conversion was low (loss ratio ≈ 0%) (Fig. 6). The VAH catalytic conversions were gradually promoted by increasing the temperature because stable aromatic compounds catalytically oxidize more predominantly at high temperatures^12,63,64,71,72^. Interestingly, in the range 250–350 °C, the VAHs with high molecular stability were not fully oxidized by the CeO_2_ catalyst (Fig. 6 and Supplementary Fig. 19c), and the conversion ratio was also different depending on the reactivity of the aromatic compounds, which can enable the gas selectivity and response toward aromatic compounds to be tailored and controlled systematically. These findings clearly suggest that the interfering gases were filtered through oxidative consumption without expending any aromatic BTEXS gases at the catalytic CeO_2_ overlayer, which enabled the exclusively high selectivity and response to volatile aromatic compounds and highlights the advantages of the proposed bilayer structure fabricated with the CeO_2_ overlayer.

### Universal sensor design for detecting volatile aromatic hydrocarbons

**Fig. 7:**
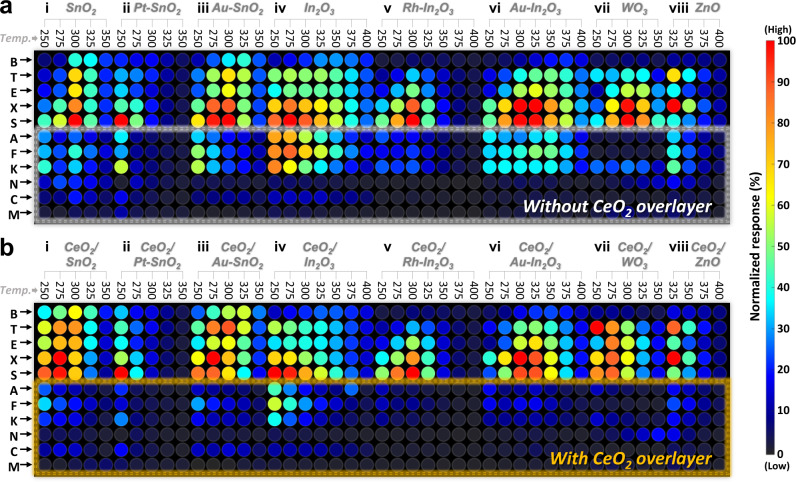
Gas-sensing patterns for diverse sensors. **a**, **b** Normalized signal intensities of diverse single-layer sensors (**a**; (i) SnO_2_, (ii) Pt–SnO_2_, (iii) Au–SnO_2_, (iv) In_2_O_3_, (v) Rh–In_2_O_3_, (vi) Au–In_2_O_3_, (vii) WO_3_, and (viii) ZnO) and CeO_2_-coated bilayer sensors (**b**; (i) 0.4CeO_2_/SnO_2_, (ii) 0.4CeO_2_/Pt–SnO_2_, (iii) 0.4CeO_2_/Au–SnO_2_, (iv) 0.4CeO_2_/In_2_O_3_, (v) 0.4CeO_2_/Rh–In_2_O_3_, (vi) 0.4CeO_2_/Au–In_2_O_3_, (vii) 0.4CeO_2_/WO_3_, and (viii) 0.4CeO_2_/ZnO) to 5 ppm analyte gases. Temp. means sensor temperature. Source data are provided as a Source Data file.

**Fig. 8:**
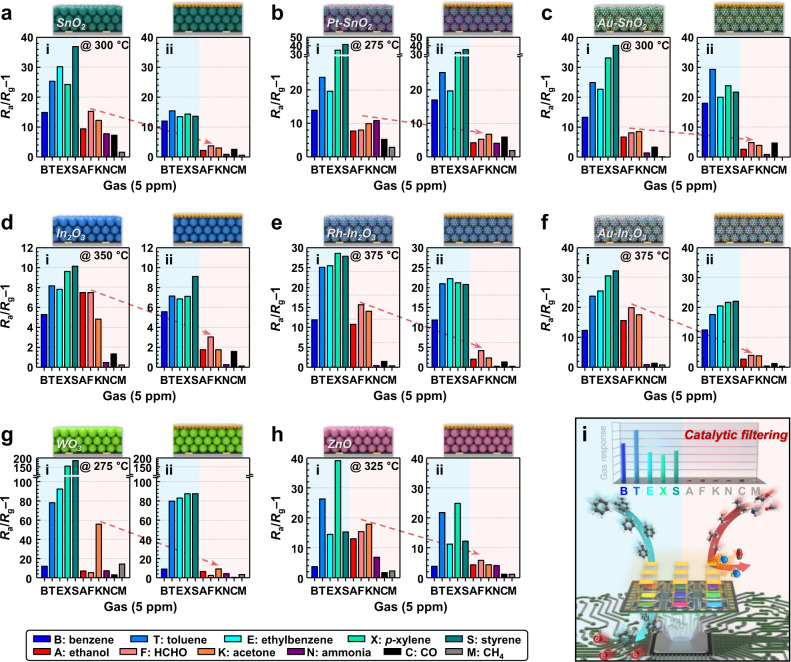
Gas responses for diverse sensors at optimal operation temperature. **a**–**h** Response properties of diverse (i) single-layer sensors and (ii) CeO_2_-coated bilayer sensors (sensing layer material: SnO_2_ (**a**), Pt–SnO_2_ (**b**), Au–SnO_2_ (**c**), In_2_O_3_ (**d**), Rh–In_2_O_3_ (**e**), Au–In_2_O_3_ (**f**), WO_3_ (**g**), and ZnO (**h**)). **i** Schematic showing the detection mechanism of the bilayer sensor. Source data are provided as a Source Data file.

The general validity for designing sensors with a CeO_2_ overlayer was investigated using different sensing materials. (Figs. 7, 8 and Supplementary Fig. 20). The diverse gas sensing materials were also prepared via ultrasonic spray pyrolysis (Supplementary Note 6). The gas-detecting characteristics are summarized in Supplementary Fig. 21, and the normalized responses are displayed in different colors according to the scale bar to facilitate comparison (Fig. 7a, b). The gas responses of the single-layer sensors (SnO_2_, Pt–SnO_2_, Au–SnO_2_, In_2_O_3_, Rh–In_2_O_3_, Au–In_2_O_3_, WO_3_, and ZnO) to the aromatic BTEXS were comparable to or slightly higher than those of the same sensors to the other gases over the entire operating-temperature range (Figs. 7a and 8a(i)–h(i), and Supplementary Fig. 21a(i)–h(i), a(ii)–h(ii)). The incomparable differences in the gas responses support again that exclusively detecting low-reactivity VAHs is difficult using a simple sensor design like those previously reported in the literature^22,33,37,42–44,51,73–78^^,S9–S23^. However, the coating and increase of the CeO_2_ overlayer thickness to 0.05, 0.1, and 0.4 μm reduced the gas response to the interfering gases, confirming the excellent batch-to-batch reproducibility. In particular, when the sensing layer was completely coated with an ~0.4-μm-thick CeO_2_ overlayer, although the responses to the nonaromatic interference gases significantly decreased over a wide temperature range, the responses to most of the BTEXS gases remained high, thereby enabling highly selective and sensitive VAH detection (Figs. 7b and 8a(ii)–h(ii), and Supplementary Fig. 21a(i)–h(i), a(iii)–h(iii)). Indeed, the CeO_2_-coated sensors exhibited considerably increased selectivities to aromatic BTEXS gases: 5.5 (0.4CeO_2_/SnO_2_ at 300 °C), 4.0 (0.4CeO_2_/Pt–SnO_2_ at 275 °C), 6.9 (0.4CeO_2_/Au–SnO_2_ at 300 °C), 3.2 (0.4CeO_2_/In_2_O_3_ at 350 °C), 5.9 (0.4CeO_2_/Rh–In_2_O_3_ at 375 °C), 4.7 (0.4CeO_2_/Au–In_2_O_3_ at 375 °C), 8.7 (0.4CeO_2_/WO_3_ at 275 °C), and 5.1 (0.4CeO_2_/ZnO at 325 °C) compared to the selectivities of the single-layer sensors fabricated without a CeO_2_ overlayer. These results confirm that the CeO_2_ overlayer can be applied to diverse sensing materials to achieve exceptional VAH selectivity by suppressing cross-responses to nonaromatic gases through oxidative filtering (Fig. 8i). Notably, not only the *R*_a_ values but also the responding (*τ*_res_) and recovering (*τ*_recov_) kinetics of the bilayer sensors were similar to those of the single-layer sensors (Supplementary Figs. 22 and 23, respectively), which enabled the gas response and selectivity to be controlled without altering the intrinsic sensor resistance and rapid sensing capability (Supplementary Note 7 and 8). Furthermore, the validity of the CeO_2_ overlayer coating on Rh–SnO_2_ sensors with different morphology (hierarchically porous Rh–SnO_2_ and Rh–SnO_2_ nanoparticles) was also evaluated (Supplementary Figs. 24). Overall, the CeO_2_-coated Rh–SnO_2_ with different morphologies showed high selectivity toward VAHs, indicating the coating of CeO_2_ overlayer can be applied to variable structured sensing materials. In this respect, these distinctive features of bilayer sensors originating from effectively separating the sensing and catalytic reactions can provide a universal and facile solution for tailoring the gas-sensing characteristics of aromatic compounds.

### The quantitative and discriminative detection of volatile aromatic compounds using bilayer sensor arrays

**Fig. 9:**
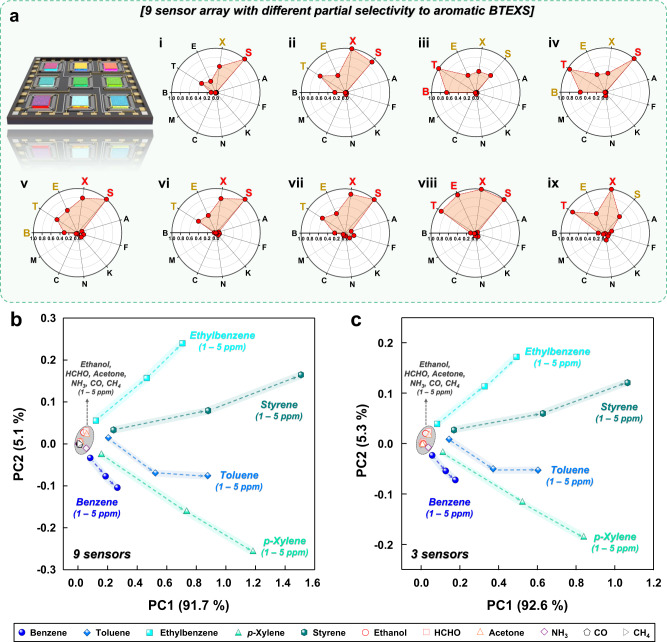
Quantitative and discriminative detection of volatile aromatic compounds using bilayer sensor arrays. **a** Polar plots of responses to 5 ppm of analyte gases for (i) 0.05CeO_2_/Rh–SnO_2_, (ii) 0.1CeO_2_/Rh–SnO_2_, (iii) 0.4CeO_2_/Rh–SnO_2_, (iv) 0.7CeO_2_/Rh–SnO_2_, (v) 0.4CeO_2_/Au–SnO_2_, (vi) 0.4CeO_2_/Rh–In_2_O_3_, (vii) 0.4CeO_2_/Au–In_2_O_3_, (viii) 0.4CeO_2_/WO_3_, and (ix) 0.4CeO_2_/ZnO sensors (B: benzene, T: toluene, E: ethylbenzene, X: *p*-xylene, S: styrene, A: ethanol, F: HCHO, K: acetone, N: ammonia, C: CO, and M: CH_4_). **b** PCA plot generated using data obtained from a 3 × 3 sensor array exhibiting different partial selectivities to demonstrate aromatic BTEXS gas discrimination over the interferences from ethanol, HCHO, acetone, ammonia, CO, and CH_4_ (concentrations: 1–5 ppm). **c** PCA result constructed by data obtained from three major sensors ((iii) 0.4CeO_2_/Rh–SnO_2_, (iv) 0.7CeO_2_/Rh–SnO_2_, (vi) 0.4CeO_2_/Rh–In_2_O_3_). Source data are provided as a Source Data file.

More importantly, the gas selectivities between the BTEXS gases could be further tuned in the bilayer sensor designs. For instance, by changing the overlayer thickness, operating temperature, and sensing material, different partial selectivities to specific aromatic gases and negligible cross-responses to interfering gases were achieved using diverse oxide chemiresistors fabricated with CeO_2_ overlayers (Fig. 9a). Because these distinctive gas selectivities can be used to discriminate different aromatic BTEXS gases, 3 × 3 multisensor arrays were assembled utilizing the 0.05CeO_2_/Rh–SnO_2_, 0.1CeO_2_/Rh–SnO_2_, 0.4CeO_2_/Rh–SnO_2_, 0.7CeO_2_/Rh–SnO_2_, 0.4CeO_2_/Au–SnO_2_, 0.4CeO_2_/Rh–In_2_O_3_, 0.4CeO_2_/Au–In_2_O_3_, 0.4CeO_2_/WO_3_, and 0.4CeO_2_/ZnO sensors, and principal component analysis (PCA) was employed to demonstrate the BTEXS gas discrimination capability (Fig. 9b and Supplementary Note 9). Interestingly, the PCA results revealed that the aromatic BTEXS were clearly separated from the nonaromatic ones and classified into five nonoverlapping clusters, even at various gas concentrations (e.g., 1, 2.5, and 5 ppm), indicating that the aromatic gases could be discriminated. To investigate the contribution of each sensor to the PCA result, we compared the PCA results by subtracting one of the nine sensors (Supplementary Fig. 25). Similar patterns with PCA plot generated from the nine sensors array were obtained even subtracting sensor (i), (ii), (v), (vii), (viii), or (ix). However, the quantitative discrimination of VAHs cannot be achieved by subtracting sensor (iii) 0.4CeO_2_/Rh–SnO_2_, (iv) 0.7CeO_2_/Rh–SnO_2_, or (vi) 0.4CeO_2_/Rh–In_2_O_3_, indicating that the contribution of the three sensors’ sensing pattern is dominant for determining the PCA results for quantitative and discriminative detection of volatile aromatic compounds (Supplementary Fig. 25c, d, f). Thus, the PCA plot similar to the sensor array consisting of nine sensors could be achieved by conducting PCA using the three major sensors (Fig. 9c). This finding has important implications because the sensors and sensor array can provide comprehensive information (e.g., gas composition and concentration) for precise and economical monitoring of aromatic gases. This finding has important implications for monitoring the health impact of aromatic gases. Moreover, the results of this study suggest that the CeO_2_-overlayer-coated sensors and sensor array are versatile, facile, and promising platforms for exclusively detecting aromatic BTEXS.

## Discussion

In the present study, we proposed an effective strategy for highly selectively and sensitively detecting traces (on the order of ppb) of volatile aromatic compounds without any interference from other indoor air pollutants. For this purpose, bilayer sensors with catalytic CeO_2_ overlayer were designed as a promising and facile sensor platform for tailoring gas-sensing characteristics. The CeO_2_-overlayer-coated Rh–SnO_2_ sensors exhibited high selectivity and responses to aromatic BTEXS, which enabled these gases to be discriminated, even in gas mixtures. PTR–QMS analysis revealed that this exceptional VAH sensing capability was attributed to the catalytic oxidation of highly reactive interfering gases to less- or nonreactive forms by the CeO_2_ overlayer, which exhibited moderate catalytic activity. Furthermore, we demonstrated the general validity of the bilayer sensor design concept by coating a CeO_2_ overlayer on the diverse sensing film using SnO_2_, Pt–SnO_2_, Au–SnO_2_, In_2_O_3_, Rh–In_2_O_3_, Au–In_2_O_3_, WO_3_, and ZnO. In addition, pattern recognition using the nine-sensor array clearly distinguished between aromatic and nonaromatic gases and quantitatively identified aromatic BTEXS. The findings of this study should lead to opportunities for designing highly precise, reliable, and cost-effective personal sensors for monitoring indoor and outdoor air qualities.

## Methods

### Synthesis of sensing materials

Rh-loaded SnO_2_ (Rh–SnO_2_) hollow spheres were synthesized using ultrasonic spray pyrolysis and subsequent calcination. Tin(II) chloride dihydrate (6.7698 g, 0.1 M), rhodium(III) chloride hydrate (0.0315 g, 0.5 at%), citric acid monohydrate (15.9197 g, 0.25 M), and diluted hydrochloric acid solution (35.0–37.0%, 6 mL) were dissolved in 300 mL of distilled water, and the solution was stirred for 1 h at room temperature (23 °C). A clear solution containing uniformly distributed Rh and Sn salts was sprayed using six ultrasonic transducers (resonance frequency: 1.7 MHz), and the droplets were transported in a carrier air gas (flow rate: 20 L min^–1^) to a high-temperature quartz reactor (700 °C). The spray pyrolysis system used in this experiment is shown in Supplementary Fig. 26a. The Rh- and Sn-containing precursor powders were both collected and annealed at 600 °C for 3 h to remove the residual carbon components and convert the powders into Rh–SnO_2_ spheres. For comparison, pure hollow SnO_2_ spheres were also synthesized using ultrasonic spray pyrolysis from an Rh-free solution. The experimental procedures for preparing the other gas-sensing materials (Pd–SnO_2_, Pt–SnO_2_, Au–SnO_2_, In_2_O_3_, Rh–In_2_O_3_, Au–In_2_O_3_, WO_3_, and ZnO hollow spheres) are described in the Supporting Information.

### Fabrication of bilayer sensor with Rh–SnO_2_ gas-sensing film and CeO_2_ overlayer

The slurry for gas-sensing-film was prepared by mixing the Rh–SnO_2_ spheres with a terpineol-based ink binder (Rh–SnO_2_/binder = 30:70 w/w) and subsequently screen-printing the slurry onto an Al_2_O_3_ substrate with two Au electrodes (1.5 mm × 1.5 mm, electrode gap: 0.2 mm) (Supplementary Fig. 26b). The sensors were heated at 450 °C for 3 h to remove any residual organic components. Subsequently, catalytic CeO_2_ overlayers were deposited onto the Rh–SnO_2_ gas-sensing films by electron-beam evaporation under vacuum (base pressure: 5 × 10^−6^ Torr) (Supplementary Fig. 26c).

## Supplementary information

Supplementary Information

## Data Availability

The data that support Figs. 2–9 can be found in the Source Data, and the data that support the findings of this study are available from the corresponding author upon request. Source data are provided with this paper.

## Source data

Source Data
