# Supplementary material for: Exclusive detection of volatile aromatic hydrocarbons using bilayer oxide chemiresistors with catalytic overlayers
Source: Nat Commun. 2023 Jan 25;14:233. doi: 10.1038/s41467-023-35916-3 (PMC9877030; doi:10.1038/s41467-023-35916-3)
Supplement: Supplementary file 1 — Supplementary Information [file 41467_2023_35916_MOESM1_ESM.pdf]

Supplementary Information

Exclusive detection of volatile aromatic hydrocarbons using bilayer oxide chemiresistors with catalytic overlayers

Seong-Yong Jeong[1,2,3,*], Young Kook Moon[1,3], Joseph Wang[2] & Jong-Heun Lee[1,4]

[1]*Department of Materials Science and Engineering, Korea University, Seoul 02841, Republic of Korea*

[2]*Department of Nanoengineering, University of California, San Diego, La Jolla, CA 92093, USA*

[3]*These authors contributed equally*

[4]*Deceased*

[*]*Corresponding author: S.-Y. Jeong*

Email: jeong7460@korea.ac.kr, s6jeong@eng.ucsd.edu

Contents

• **Supplementary Notes**.. 5–14

- **Supplementary Note 1 |** Supplementary experimental section (Materials, Characterization of materials & Gas-sensing measurements) 5

- **Supplementary Note 2 |** Optimization of the sensing materials.......................................7

- **Supplementary Note 3 |** Gas selectivity comparison..8

- **Supplementary Note 4 |** Multi-component gas-sensing analysis 9

- **Supplementary Note 5 |** Catalytic evaluation 10

- **Supplementary Note 6 |** Synthesis of supplementary sensing materials 11

- **Supplementary Note 7 |** Comparison of sensor resistances..12

- **Supplementary Note 8 |** Comparison of response and recovery times...........................13

- **Supplementary Note 9 |** Principal components analysis (PCA) for pattern recognition..14

• **Supplementary Figures** 15–41

- **Supplementary Fig. 1 |** N_2 adsorption/desorption isotherms and pore-size distribution of Rh–SnO_2 spheres...15

- **Supplementary Fig. 2 |** HR-TEM image of Rh–SnO_2 sphere..16

- **Supplementary Fig. 3 |** SEM image and XRD pattern of CeO_2 thin film deposited on Si substrate 17

- **Supplementary Fig. 4 |** Sn 3*d* and Rh 3*d* XPS spectra for CeO_2/Rh–SnO_2 film 18

- **Supplementary Fig. 5 |** SEM, TEM, and EDS mapping images and XRD pattern of SnO_2 hollow spheres...19

- **Supplementary Fig. 6 |** Dynamic sensing transients of pure SnO_2, Rh–SnO_2, and 0.4CeO_2/Rh–SnO_2 sensors 20

- **Supplementary Fig. 7 |** O1*s* XPS spectra generated for SnO_2 and Rh–SnO_2 21

- **Supplementary Fig. 8 |** SEM, TEM, and EDS mapping images of Pd–SnO_2, Pt–SnO_2, and Au–SnO_2 hollow spheres..22

- **Supplementary Fig. 9 |** Gas-sensing characteristics and responses of Pd–SnO_2, Pt–SnO_2, Au–SnO_2, and Rh–SnO_2 sensors 23

- **Supplementary Fig. 10 |** Cross-sectional SEM images of 0.05CeO_2/Rh–SnO_2, 0.1CeO_2/Rh–SnO_2, and 0.7CeO_2/Rh–SnO_2 films 24

- **Supplementary Fig. 11 |** Gas-sensing characteristics and responses of 0.05CeO_2/Rh–SnO_2, 0.1CeO_2/Rh–SnO_2, and 0.7CeO_2/Rh–SnO_2 sensors 25

- **Supplementary Fig. 12 |** VAH selectivity (S_{VAH}/S_A) comparison of SnO_2, Rh–SnO_2, 0.05CeO_2/Rh–SnO_2, 0.1CeO_2/Rh–SnO_2, 0.4CeO_2/Rh–SnO_2, and 0.7CeO_2/Rh–SnO_2 sensors 26

- **Supplementary Fig. 13 |** VAH responses of the sensors in each batch (SnO_2, Rh–SnO_2, 0.4CeO_2/Rh–SnO_2, 0.05CeO_2/Rh–SnO_2, 0.1CeO_2/Rh–SnO_2, and 0.7CeO_2/Rh–SnO_2 27

- **Supplementary Fig. 14 |** Sensor measurement in simulated condition (relative humidity: 50% at 18 °C). 28

- **Supplementary Fig. 15 |** Seven repeated sensing transients and long-term stabilities of 0.4CeO_2/Rh–SnO_2 sensor 29

- **Supplementary Fig. 16 |** Multi-component gas-sensing analysis......30

- **Supplementary Fig. 17 |** Gas responses to aromatic compounds plotted as functions of CeO_2 overlayer thickness for Rh–SnO_2, 0.05CeO_2/Rh–SnO_2, 0.1CeO_2/Rh–SnO_2, 0.4CeO_2/Rh–SnO_2, and 0.7CeO_2/Rh–SnO_2 sensors in range 250–350 °C......31

- **Supplementary Fig. 18 |** EPR spectrum of CeO_2 32

- **Supplementary Fig. 19 |** Catalytic performance of CeO_2 measured using PTR-QMS 33

- **Supplementary Fig. 20 |** Cross-sectional SEM and EPMA elemental mapping images of 0.4CeO_2/SnO_2, 0.4CeO_2/Pt–SnO_2, 0.4CeO_2/Au–SnO_2, 0.4CeO_2/In_2O_3, 0.4CeO_2/Rh–In_2O_3, 0.4CeO_2/Au–In_2O_3, 0.4CeO_2/WO_3, and 0.4CeO_2/ZnO films 34

- **Supplementary Fig. 21 |** VAH selectivity (S_{VAH}/S_A) and gas-sensing properties of $x$$CeO_2$/$SnO_2$, $x$$CeO_2$/Pt–$SnO_2$, $x$$CeO_2$/Au–$SnO_2$, $x$$CeO_2$/$In_2O_3$, $x$$CeO_2$/Rh–$In_2O_3$, $x$$CeO_2$/Au–$In_2O_3$, $x$$CeO_2$/$WO_3$, and $x$$CeO_2$/ZnO sensors 35

- **Supplementary Fig. 22 |** Sensor resistances (R_a) of $x$$CeO_2$/Rh–$SnO_2$, $x$$CeO_2$/$SnO_2$, $x$$CeO_2$/Pt–$SnO_2$, $x$$CeO_2$/Au–$SnO_2$, $x$$CeO_2$/$In_2O_3$, $x$$CeO_2$/Rh–$In_2O_3$, $x$$CeO_2$/Au–$In_2O_3$, $x$$CeO_2$/$WO_3$, and $x$$CeO_2$/ZnO sensors 36

- **Supplementary Fig. 23 |** Ninety percent (90%) response and recovery times (τ_{res} and τ_{recov}, respectively) of $x$$CeO_2$/Rh–$SnO_2$, $x$$CeO_2$/$SnO_2$, $x$$CeO_2$/Pt–$SnO_2$, $x$$CeO_2$/Au–$SnO_2$, $x$$CeO_2$/$In_2O_3$, $x$$CeO_2$/Rh–$In_2O_3$, $x$$CeO_2$/Au–$In_2O_3$, $x$$CeO_2$/$WO_3$, and $x$$CeO_2$/ZnO sensors..37

- **Supplementary Fig. 24 |** SEM images of the hierarchically porous and the nanoparticle Rh–SnO_2, and gas sensing characteristics of CeO_2/hierarchically porous Rh–SnO_2 and CeO_2/nanoparticle Rh-SnO_2 sensors 38

- **Supplementary Fig. 25 |** PCA results constructed by gas response patterns of the 8 sensors subtracted from the 9 sensors array......39

- **Supplementary Fig. 26 |** Schematic of overall experimental procedure (spray pyrolysis; screen printing; and e-beam evaporation)......40

- **Supplementary Fig. 27 |** Schematic of gas-sensor measurement system 41

• **Supplementary Table** 42–45

- **Supplementary Table 1 |** Properties of various materials used for sensing gaseous volatile aromatic hydrocarbons, as reported in literature and obtained in this study...........36

• **References** 46–48

Supplementary Note 1 | Supplementary experimental section (Materials, Characterization of materials & Gas-sensing measurements).

Materials. Tin(II) chloride dihydrate ($SnCl_2·2H_2O$, ≥ 98%, Sigma–Aldrich, USA), rhodium(III) chloride hydrate ($RhCl_3·xH_2O$, ≥ 99.9%, Sigma–Aldrich, USA), gold(III) chloride trihydrate ($HAuCl_4·3H_2O$, ≥ 99.9%, Sigma–Aldrich, USA), citric acid monohydrate ($C_6H_8O_7·H_2O$, ≥ 99.0%, Sigma–Aldrich, USA), and diluted hydrochloric acid solution (HCl, 35.0–37.0%, Samchun Chemical Co., Ltd., Korea) were used without further purification for fabricating the gas-sensing materials. An ink vehicle (terpineol-based binder, FCM, USA) was used to fabricate the screen-printed gas-sensing films. Cerium(IV) oxide grains (99.9%, Kojundo Chemical Laboratory Co., Ltd., Japan) were used to prepare the catalytic CeO_2 overlayer by e-beam evaporation.

Characterization of materials. Field-emission scanning electron microscopy (FE-SEM, SU-70, Hitachi Co., Ltd., Japan) and high-resolution transmission electron microscopy (HR-TEM, JEM-ARM200F, JEOL, Co., Ltd., Japan) were used to observe the morphology and microstructure of the synthesized materials and gas-sensing films, respectively. The pore size distribution and specific surface area of the Rh–SnO_2 powder were analyzed using Brunauer–Emmett–Teller (BET) analysis of nitrogen adsorption isotherms (TriStar 3000, Micromeritics, USA). Field-emission electron-probe microanalysis (FE-EPMA, JXA-8530F, JEOL Co., Ltd., Japan) was used to obtain elemental mappings of the bilayer sensors. The phase and crystallinity of the bilayer gas-sensing films were characterized using X-ray diffraction (XRD, D/MAX-2500 V/PC, Rigaku, Japan) equipped with a CuKα radiation source (λ = 1.5418 Å). The

chemical states of the bilayer films were analyzed using X-ray photoelectron spectroscopy (XPS, PHI X-tool, ULVAC-PHI, Japan).

Gas-sensing measurements. Prior to the gas-sensing measurements, the as-prepared CeO_2-coated Rh–SnO_2 bilayer sensors were stabilized by annealing at 450 °C for 2 h. The sensors were covered with a Ni-plated Kovar housing cap and stainless-steel gauze and enclosed in a specially designed acetal chamber. Gas-sensing measurements were conducted in dry air through interval exposure to analyte gases. The atmospheric conditions (dry air or analyte gas) were controlled using mass flow controllers and an automatic four-way valve to ensure a constant gas flow rate of 300 $cm^3 \cdot min^{-1}$. The concentrations of analyte gases were independently controlled by mixing ratios between a synthetic gases and dry air. The test chamber was cleaned by fluxing dry air prior to sensor measurements. The sensor resistance was measured using a digital multimeter (DMM6500, Keithley, Tektronix, Inc., USA) equipped with a 10-channel multiplexer (Model 2000-SCAN scanner card, Keithley, Tektronix, Inc., USA), and the sensor temperature was controlled by applying voltage to the Ru microheater formed at the bottom of the sensor substrate using a direct current (DC) power supply (2230-30-1, Keithley, Tektronix, Inc., USA) (Supplementary Fig. 27).

Supplementary Note 2 | Optimization of the sensing materials.

To verify the unique role of the Rh catalyst, SnO_2 spheres were prepared using different noble metals (Pd, Pt, and Au), and their gas-sensing characteristics were compared with those of the Rh–SnO_2 sensor at elevated temperatures (250–350 °C) (Supplementary Figs. 8 and 9). The Pd–SnO_2 sensor exhibited low responses to all the analyte gases (S = 1.1–6.2 at 300 °C) (Supplementary Fig. 9a, e), which hindered VAH detection. In contrast, coating the SnO_2 surfaces with Pt or Au catalysts slightly increased the gas responses probably because of the electronic and chemical promotions (Supplementary Fig. 9b, c, f, g)[S1,S2], whereas the response was enhanced substantially less than that of the Rh–SnO_2 sensor (Fig. 3b and Supplementary Fig. 9d,h), indicating that coating the SnO_2 spheres with Rh is an effective method for enhancing the analyte gas responses.

Supplementary Note 3 | Gas selectivity comparison.

The VAH gas selectivity (S_{VAH}/S_A) was calculated based on the ratio of the most stable benzene response to the key interfering ethanol response, and the results are plotted as functions of the sensing temperature (Supplementary Fig. 12). All the S_{VAH}/S_A values of the SnO_2 and Rh–SnO_2 sensors were low and similar. In contrast, the S_{VAH}/S_A values drastically increased when the Rh–SnO_2 gas-sensing film was coated with the CeO_2 overlayer, suggesting the potential for tailoring VAH selectivity by modulating the catalytic overlayer.

Supplementary Note 4 | Multi-component gas-sensing analysis.

The distinctive features (Fig. 4 and Supplementary Fig. 14) of the bilayer sensor make it suitable for practical applications. Ethanol, HCHO, and acetone are the major indoor pollutants produced by cleaning supplies, alcoholic beverages, culinary use, and resin decomposition[S3,S4]. Ammonia is another representative interfering gas that is not only generated by meat tissues during storage and cooking but also emitted by the chemical industry[S5,S6]. CO and CH_4 are well-known indoor pollutants generated by the incomplete combustion of gases or fuels[S7,S8]. Notably, these six interfering gases are ubiquitous, and most oxide chemiresistors exhibit higher responses to these gases than to aromatic compounds, which may cause gas alarms to malfunction. Therefore, the selective detection properties of the Rh–SnO_2 and 0.4CeO_2/Rh–SnO_2 sensors were evaluated by comparing the gas responses to 5 ppm of pure BTEXS and BTEXS-containing gas mixtures (comprising 5 ppm of BTEXS and 1–5 ppm of interfering gas) (Supplementary Fig. 14a). As shown in Fig. 4 and Supplementary Fig. 14, the Rh–SnO_2 sensor detection characteristics considerably fluctuated depending on the gas concentration, whereas the 0.4CeO_2/Rh–SnO_2 sensor exhibited well-defined BTEXS gas detection characteristics, even when the gas mixture contained a high interfering-gas concentration, suggesting that these sensors offer a highly reliable and precise solution for selectively detecting aromatic BTEXS gases. To the best of our knowledge, this is the first study wherein aromatic BTEXS vapors were highly selectively and sensitively detected with negligible cross-responses to other representative indoor pollutants such as ethanol, HCHO, acetone, ammonia, CO, and CH_4.

Supplementary Note 5 | Catalytic evaluation.

The analyte gas conversions (η) were calculated using Equation (S1) as follows:

$$H = 1 - [C]_{out}/[C]_{in}) \times 100(\%), \quad \text{(S1)}$$

where $[C]_{in}$ and $[C]_{out}$ are the gas concentrations before and after the reaction at elevated temperatures from 100 to 475 °C, respectively.

The CeO_2-induced catalytic oxidation of VAHs and interference gases was investigated using proton transfer reaction–quadrupole mass spectrometry (PTR–QMS 300, Ionicon Analytik GmbH, Austria) (Supplementary Fig. 17). For this, CeO_2 powder (0.1 g) was loaded onto a quartz support and placed in the middle of a tubular quartz reactor (length: 400 mm, inner diameter: 8 mm) in a tube furnace. The reactant gas flow rate was maintained at 200 $cm^3 \cdot min^{-1}$. The concentrations of the outlet gas were determined using an online PTR–QMS. The drift tube conditions were fixed at 600 V, 80 °C, and 2.3 mbar, and the electric field strength/gas number density ratio (E/N) was set to 136 Td (1 Td = 10^{-17} V cm^2). H_3O^+ was used as the primary ion.

Supplementary Note 6 | Synthesis of supplementary sensing materials.

Pd–SnO_2, Pt–SnO_2, Au–SnO_2, In_2O_3, Rh–In_2O_3, Au–In_2O_3, WO_3, and ZnO hollow spheres were obtained by ultrasonic spray pyrolysis and subsequently heated. Briefly, the aqueous solutions used for preparing Pd–SnO_2 [0.0347 g of $Pd(NO_3)_2{\cdot}xH_2O$, 6.7698 g of $SnCl_2{\cdot}2H_2O$, 15.9197 g of $C_6H_8O_7$, and 6 mL of diluted hydrochloric acid solution], Pt–SnO_2 (0.0618 g of H_2PtCl_6, 6.7698 g of $SnCl_2{\cdot}2H_2O$, 15.9197 g of $C_6H_8O_7$, and 6 mL of diluted hydrochloric acid solution), Au–SnO_2 (0.0512 g of $HAuCl_4{\cdot}xH_2O$, 6.7698 g of $SnCl_2{\cdot}2H_2O$, 15.9197 g of $C_6H_8O_7$, and 6 mL of diluted hydrochloric acid solution), In_2O_3 [4.5125 g of $In(NO_3)_3{\cdot}xH_2O$ and 15.4809 g of $C_{12}H_{22}O_{11}$], Rh–In_2O_3 [0.0158 g of $RhCl_3{\cdot}xH_2O$, 4.5125 g of $In(NO_3)_3{\cdot}xH_2O$, and 15.4809 g of $C_{12}H_{22}O_{11}$], Au–In_2O_3 [0.0256 g of $HauCl_4{\cdot}xH_2O$, 4.5125 g of $In(NO_3)_3{\cdot}xH_2O$, and 15.4809 g of $C_{12}H_{22}O_{11}$], WO_3 [1.4194 g of WO_3, 3.1839 g of $C_6H_8O_7$, 30.0 g of $H(OCH_2CH_2)_nOH$, and 30 mL of 28.0–30.0% diluted NH_4OH solution], and ZnO hollow spheres [11.3641 g of $Zn(NO_3)_2{\cdot}xH_2O$ and 6.3679 g of $C_6H_8O_7$] were dissolved in 300 mL of distilled water. The solutions were atomized into droplets using ultrasonic transducers, and the droplets were subsequently passed through a high-temperature tubular reactor (Pd–SnO_2: 700 °C, Pt–SnO_2: 700 °C, Au–SnO_2: 700 °C, In_2O_3: 900 °C, Rh–In_2O_3: 900 °C, Au–In_2O_3: 900 °C, WO_3: 700 °C, and ZnO: 700 °C) by air at flow rate of 20 L min^{-1}. The precursor powders were collected using a Teflon™ bag filter and converted to Pd–SnO_2, Pt–SnO_2, Au–SnO_2, In_2O_3, Rh–In_2O_3, Au–In_2O_3, WO_3, and ZnO hollow spheres, respectively, by heating at 600 °C for 3 h in air.

Supplementary Note 7 | Comparison of sensor resistances.

The sensor R_a values were compared to emphasize the unique bilayer sensor design advantages (Supplementary Fig. 22). Notably, the intrinsic sensor R_a values were maintained despite the CeO_2 overlayer coating, indicating that conduction was hardly affected by the outlying CeO_2 overlayer along the lower part of the gas-sensing film near the Au electrodes. This might be because the distinctive bilayer design separated the sensing and catalytic reactions into independent processes, which enabled both the gas response and selectivity to be tailored without altering the sensor resistance. Therefore, the proposed CeO_2-overlayer-coated sensors are versatile, facile, economical, and promising platforms for controlling gas responses and selectivities without affecting sensor resistances.

Supplementary Note 8 | Comparison of response and recovery times.

The response and recovery kinetics (τ_{res} and τ_{recov}: the times required to reach 90% of the resistance variation when a sensor is exposed to 5 ppm of benzene and ambient air, respectively) of the single-layer and CeO_2-coated bilayer sensors were further calculated based on the sensing transients (Supplementary Fig. 23). Interestingly, the τ_{res} and τ_{recov} values of the $0.4CeO_2$/Rh–SnO_2 sensors (4–7 and 137–239 s, respectively) were comparable to those of the Rh–SnO_2 sensor for benzene detection. In addition, the $0.4CeO_2/SnO_2$, $0.4CeO_2$/Pt–SnO_2, $0.4CeO_2$/Au–SnO_2, $0.4CeO_2/In_2O_3$, $0.4CeO_2$/Rh–In_2O_3, $0.4CeO_2$/Au–In_2O_3, $0.4CeO_2/WO_3$, and $0.4CeO_2$/ZnO bilayer sensors exhibited similar rapid response and recovery kinetics (τ_{res} and τ_{recov}) of 4 and 939, 9 and 322, 8 and 403, 5 and 341, 5 and 294, 6 and 1636, 2 and 5863, and 4 and 1642 s, respectively, suggesting the possibility for rapidly monitoring aromatic compounds in real time. The VAHs were rapidly detected because the nanothin CeO_2 overlayer coating did not deteriorate the VAH sensing response and recovery kinetics.

Supplementary Note 9 | Principal components analysis (PCA) for pattern recognition.

The principal components analysis (PCA) was used for pattern recognition to classify multivariant sensor data. PCA is a method of finding the axis that best describes all variables by obtaining the covariance matrix and performing eigenvalue decomposition. A data matrix for single analyte was constructed by gas response data from nine sensors ((i) 0.05CeO_2/Rh–SnO_2, (ii) 0.1CeO_2/Rh–SnO_2, (iii) 0.4CeO_2/Rh–SnO_2, (iv) 0.7CeO_2/Rh–SnO_2, (v) 0.4CeO_2/Au–SnO_2, (vi) 0.4CeO_2/Rh–In_2O_3, (vii) 0.4CeO_2/Au–In_2O_3, (viii) 0.4CeO_2/WO_3, and (ix) 0.4CeO_2/ZnO) for 1–5 ppm of B: benzene, T: toluene, E: ethylbenzene, X: *p*-xylene, S: styrene, A: ethanol, F: HCHO, K: acetone, N: ammonia, C: CO, and M: CH_4. From this matrix, the eigenvalue and eigenvector were calculated. Each observation was projected onto this eigenvector line to get a coordinate value along the principal component line. The first principal component (PC1) represents the maximum variance direction in the data. A second PC (PC2) exhibits the second-largest variance along the direction orthogonal to PC1. After calculating the third PC (PC3: orthogonal to both PC1 and PC2), the percentages of PC1, PC2, and PC3 out of the total component (PC1+PC2+PC3) were calculated. Since the sum between PC1 and PC2 was sufficiently high (> 90%), PC1 and PC2 were plotted in Fig. 9.

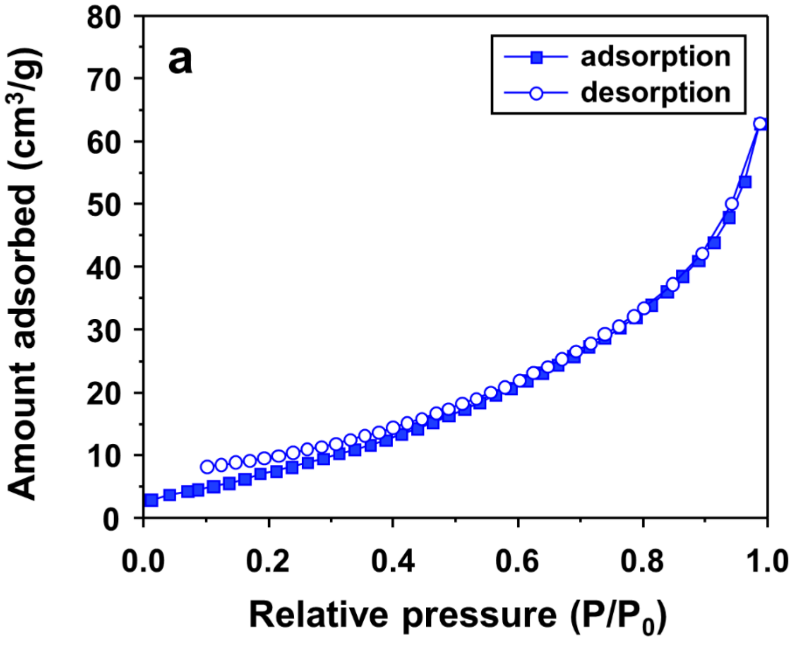

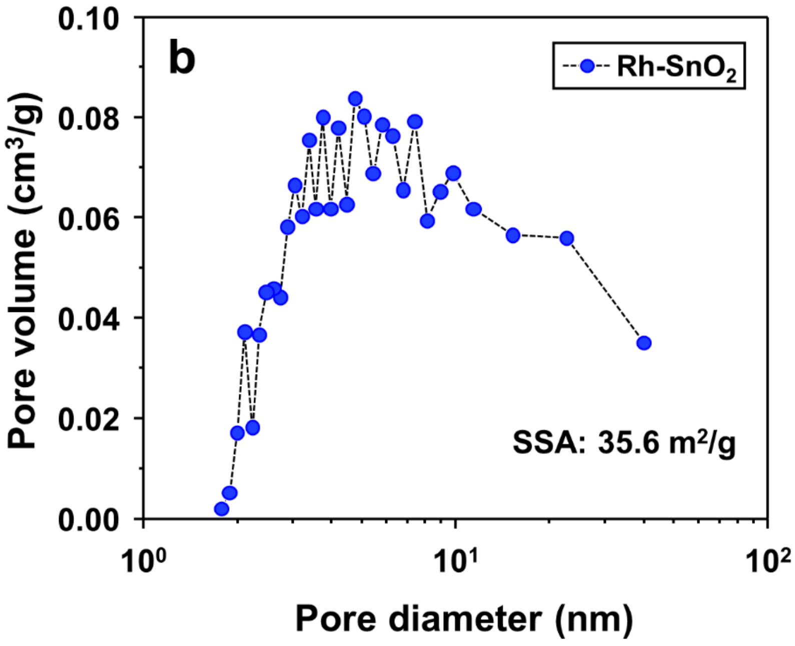

Supplementary Fig. 1 | a–b N_2 adsorption/desorption isotherms (**a**) and pore-size distribution of Rh–SnO_2 spheres (**b**) (SSA: BET specific surface area).

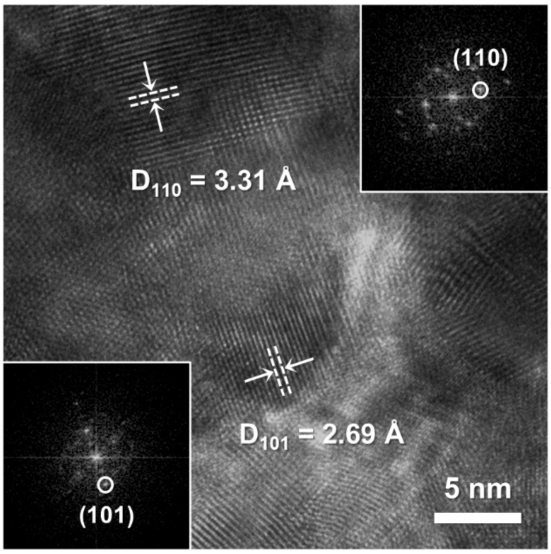

Supplementary Fig. 2 | HR-TEM image of Rh–SnO_2 sphere.

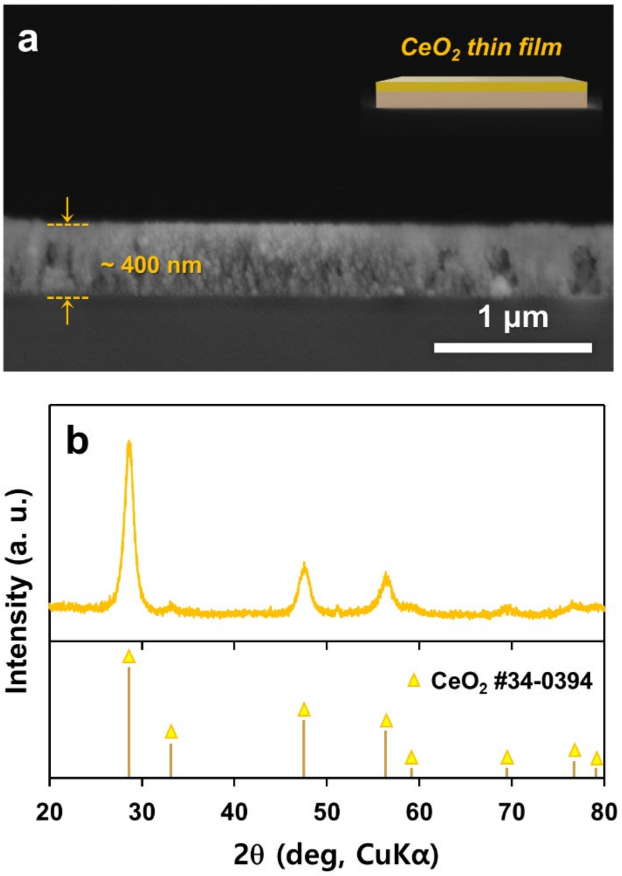

Supplementary Fig. 3 | a–b SEM image (**a**) and XRD pattern (**b**) of CeO_2 thin film deposited on Si substrate.

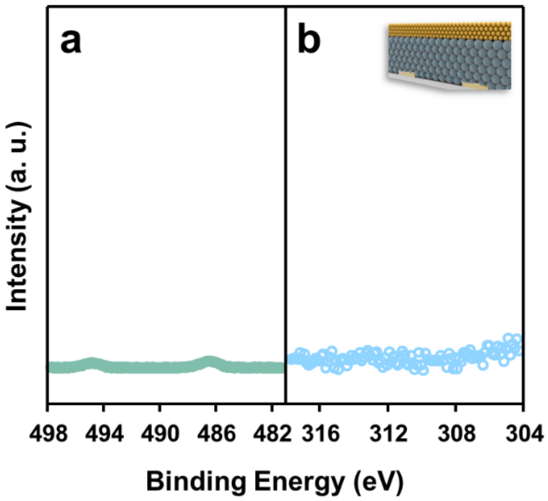

Supplementary Fig. 4 | a–b Sn 3*d* (**a**) and Rh 3*d* (**d**) XPS spectra for CeO_2/Rh–SnO_2 film.

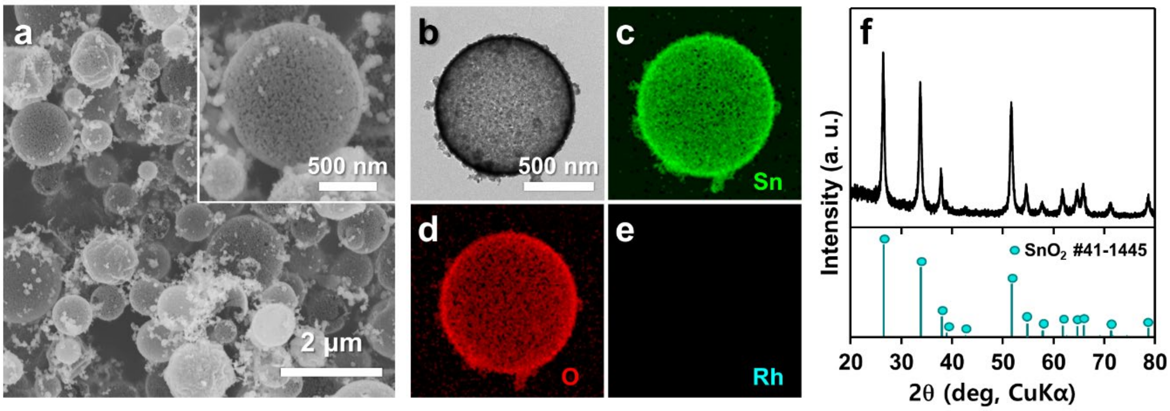

Supplementary Fig. 5 | a–f SEM (**a**), TEM (**b**), and EDS mapping (**c–e**) images and XRD pattern (**f**) of SnO_2 hollow spheres.

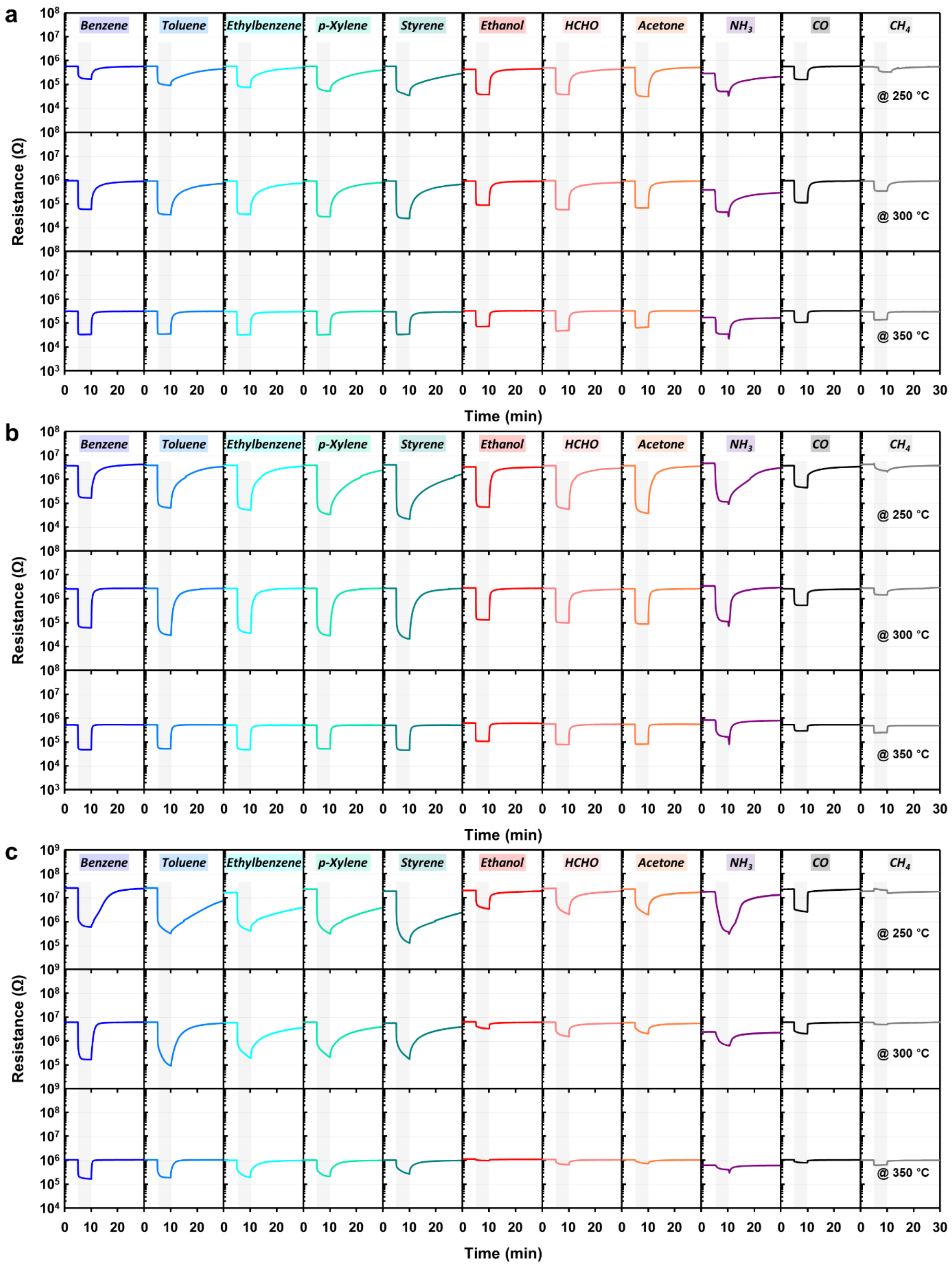

Supplementary Fig. 6 | a–c Dynamic sensing transients of pure SnO_2 (**a**), Rh–SnO_2 (**b**), and 0.4CeO_2/Rh–SnO_2 (**c**) sensors (analyte gas concentration: 5 ppm; temperature range: 250–350 °C).

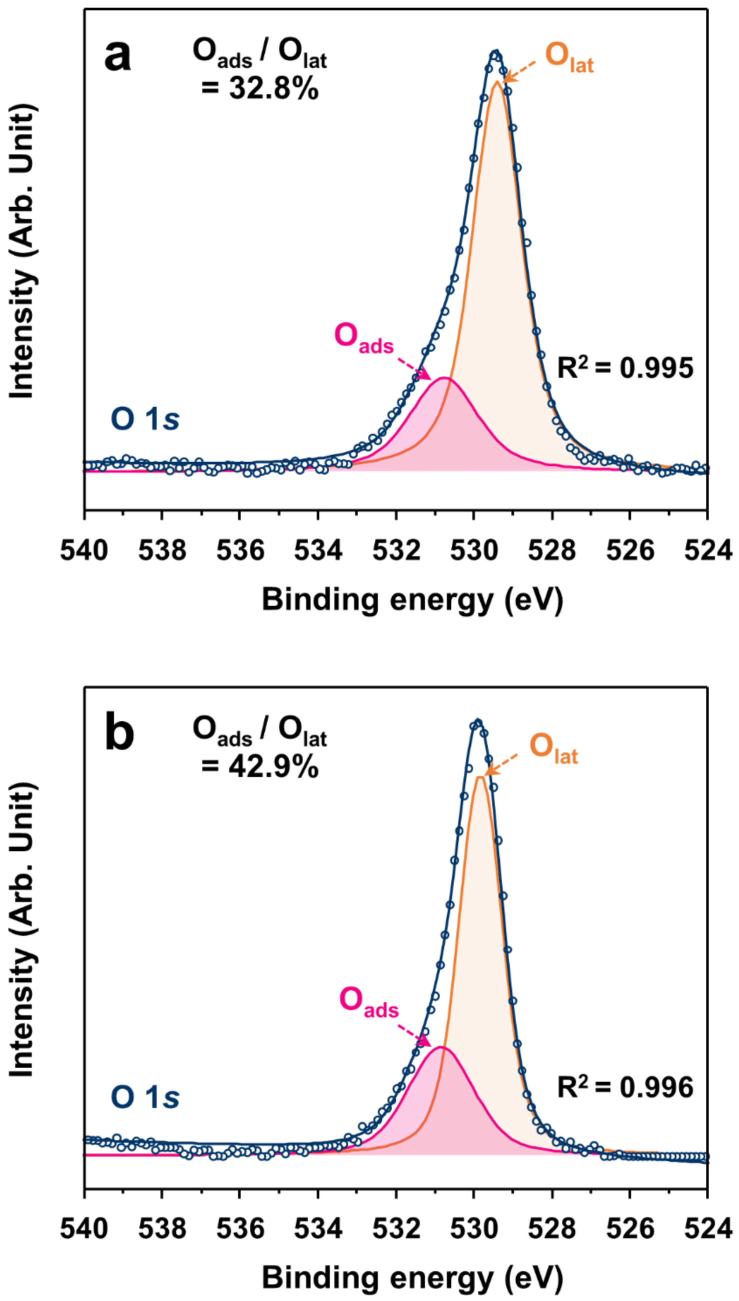

Supplementary Fig. 7 | a–b O1*s* XPS spectra generated for SnO_2 (R-squared value: 0.995) (**a**) and Rh–SnO_2 (R-squared value: 0.996) (**b**) (O_{ads}; adsorbed oxygen, O_{lat}; lattice oxygen).

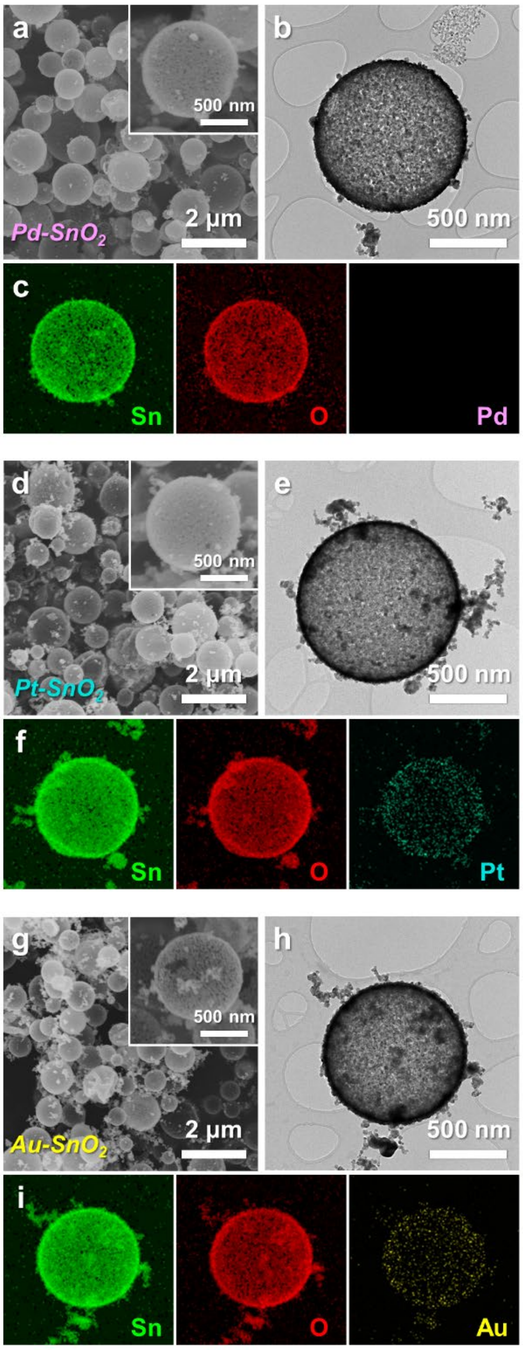

Supplementary Fig. 8 | a–i SEM, TEM, and EDS mapping images of Pd–SnO_2 (**a**–**c**), Pt–SnO_2 (**d**–**f**), and Au–SnO_2 (**g**–**i**) hollow spheres.

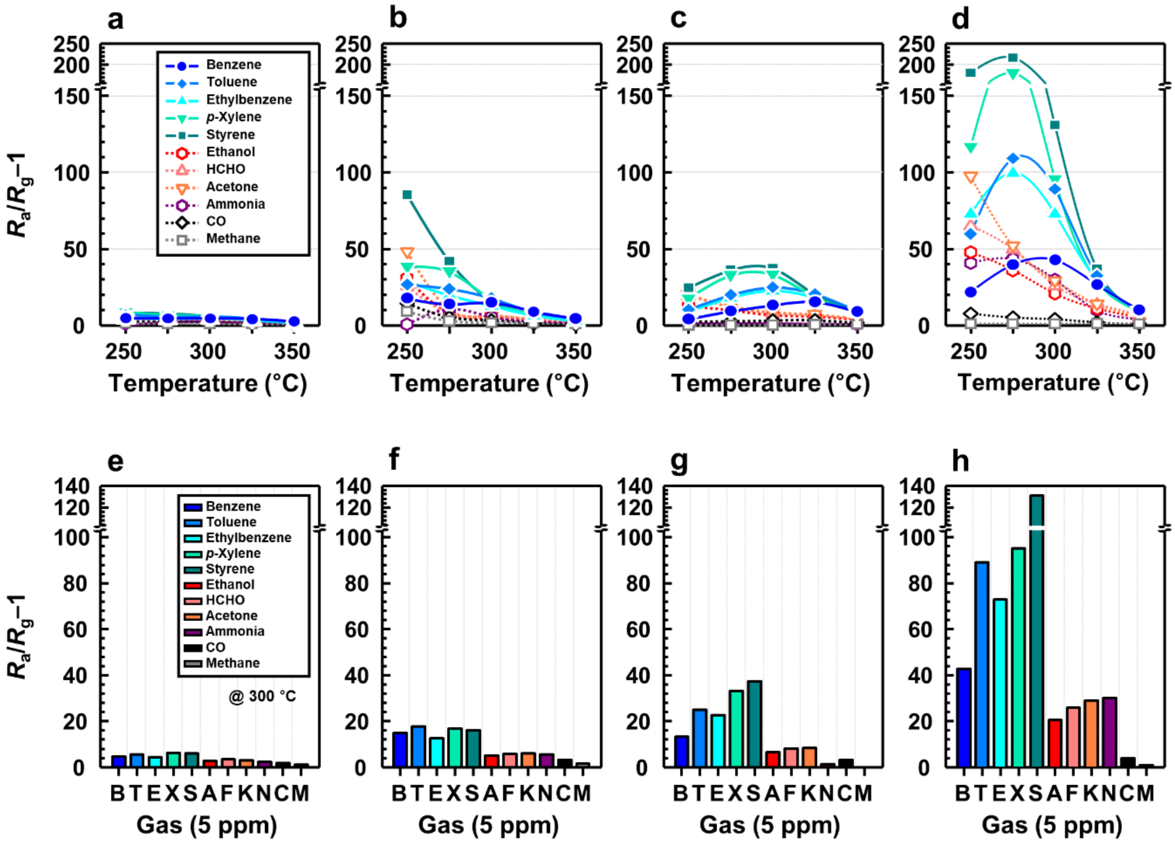

Supplementary Fig. 9 | a–h Gas-sensing characteristics and responses of Pd–SnO_2 (**a** and **e**), Pt–SnO_2 (**b** and **f**), Au–SnO_2 (**c** and **g**), and Rh–SnO_2 (**d** and **h**) sensors (analyte gas concentration: 5 ppm; temperature range: 250–350 °C).

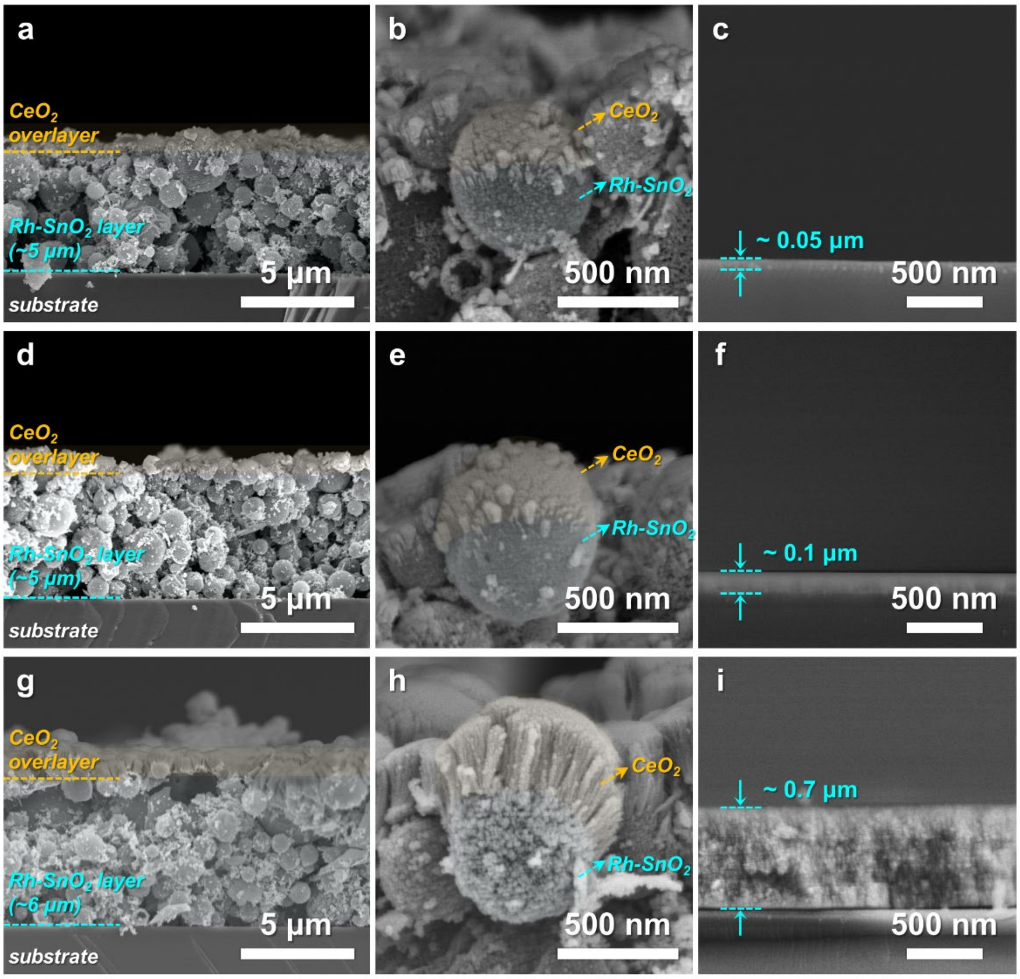

Supplementary Fig. 10 | a–h Low- and high-magnification cross-sectional SEM images of 0.05CeO_2/Rh–SnO_2 (**a** and **b**), 0.1CeO_2/Rh–SnO_2 (**d** and **e**), and 0.7CeO_2/Rh–SnO_2 (**g** and **h**) films. **c–i** Cross-sectional SEM images of 0.05-μm-thick CeO_2 (**c**), 0.1-μm-thick CeO_2 (**f**), and 0.7-μm-thick CeO_2 (**i**) thin films deposited on Si substrates.

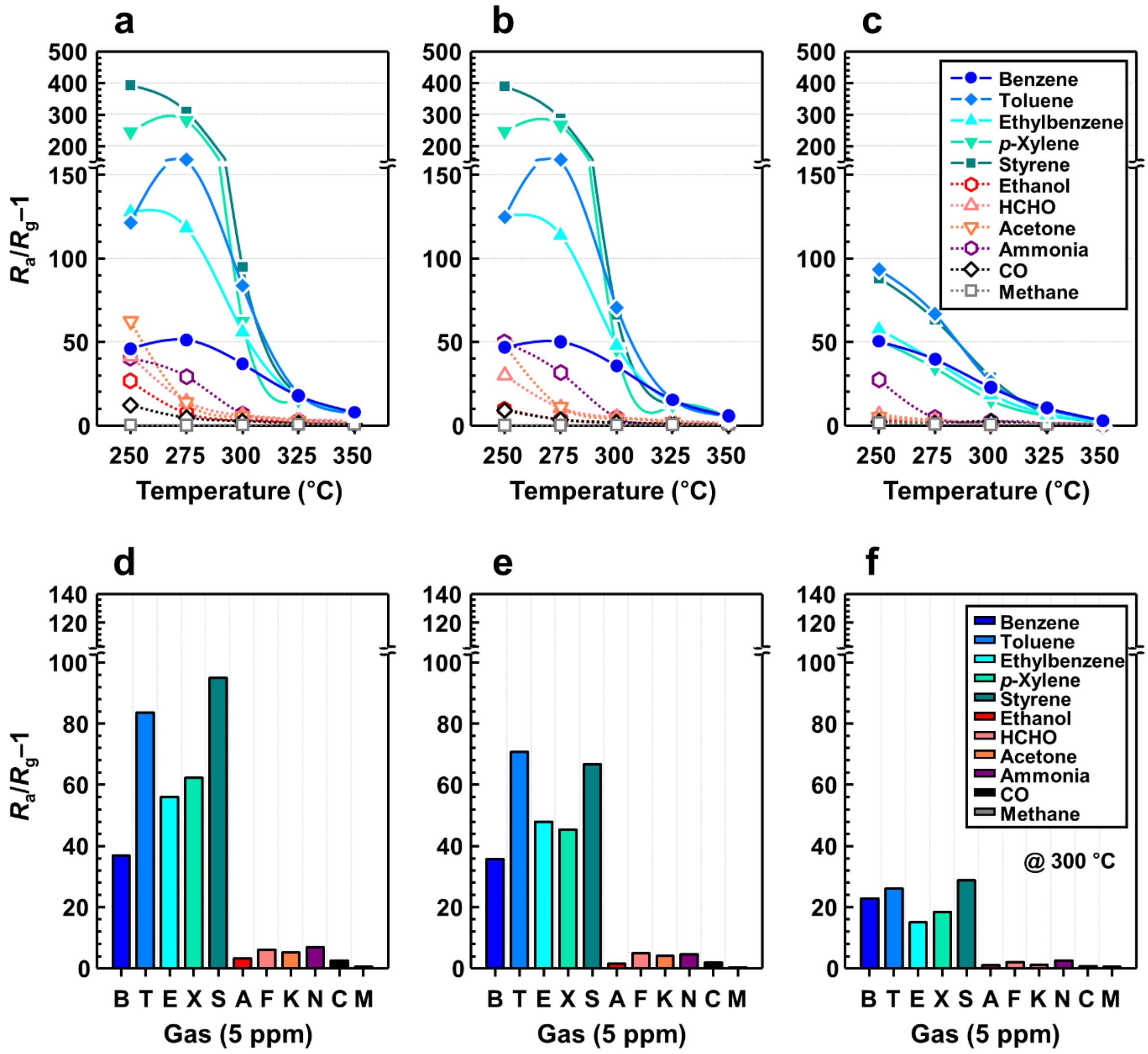

Supplementary Fig. 11 | a–f Gas-sensing characteristics and responses of $0.05CeO_2/Rh–SnO_2$ (**a** and **d**), $0.1CeO_2/Rh–SnO_2$ (**b** and **e**), and $0.7CeO_2/Rh–SnO_2$ (**c** and **f**) sensors (analyte gas concentration: 5 ppm; temperature range: 250–350 °C).

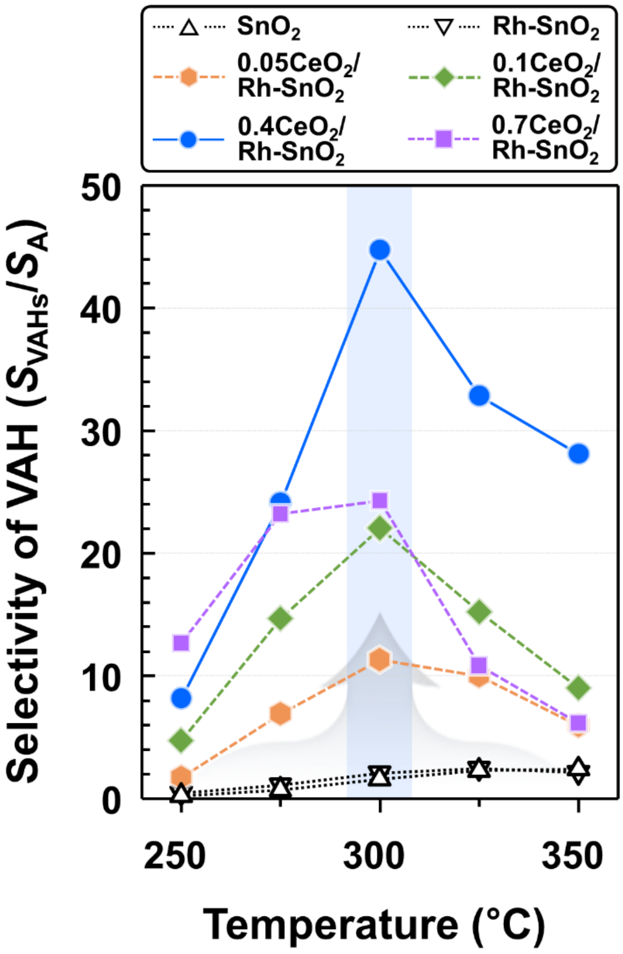

Supplementary Fig. 12 | VAH selectivity (S_{VAH}/S_A) of SnO_2, Rh–SnO_2, 0.05CeO_2/Rh–SnO_2, 0.1CeO_2/Rh–SnO_2, 0.4CeO_2/Rh–SnO_2, and 0.7CeO_2/Rh–SnO_2 sensors (analyte gas concentration: 5 ppm; temperature range: 250–350 °C).

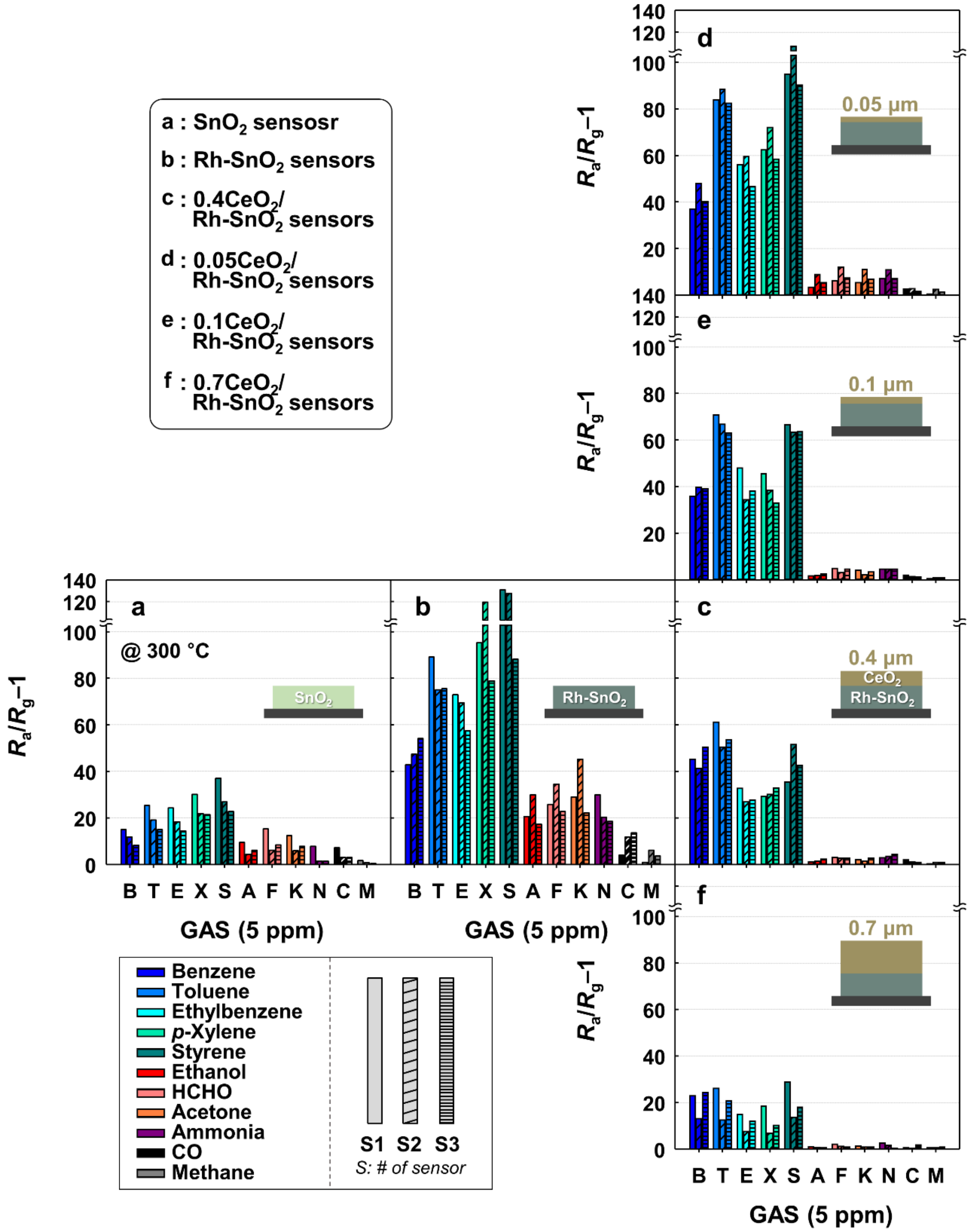

Supplementary Fig. 13 | a–f VAH responses of the sensors in each batch (SnO_2 (**a**), Rh–SnO_2 (**b**), 0.4CeO_2/Rh–SnO_2 (**c**), 0.05CeO_2/Rh–SnO_2 (**d**), 0.1CeO_2/Rh–SnO_2 (**e**), and 0.7CeO_2/Rh–SnO_2 (**f**); analyte gas concentration: 5 ppm; temperature range: 300 °C).

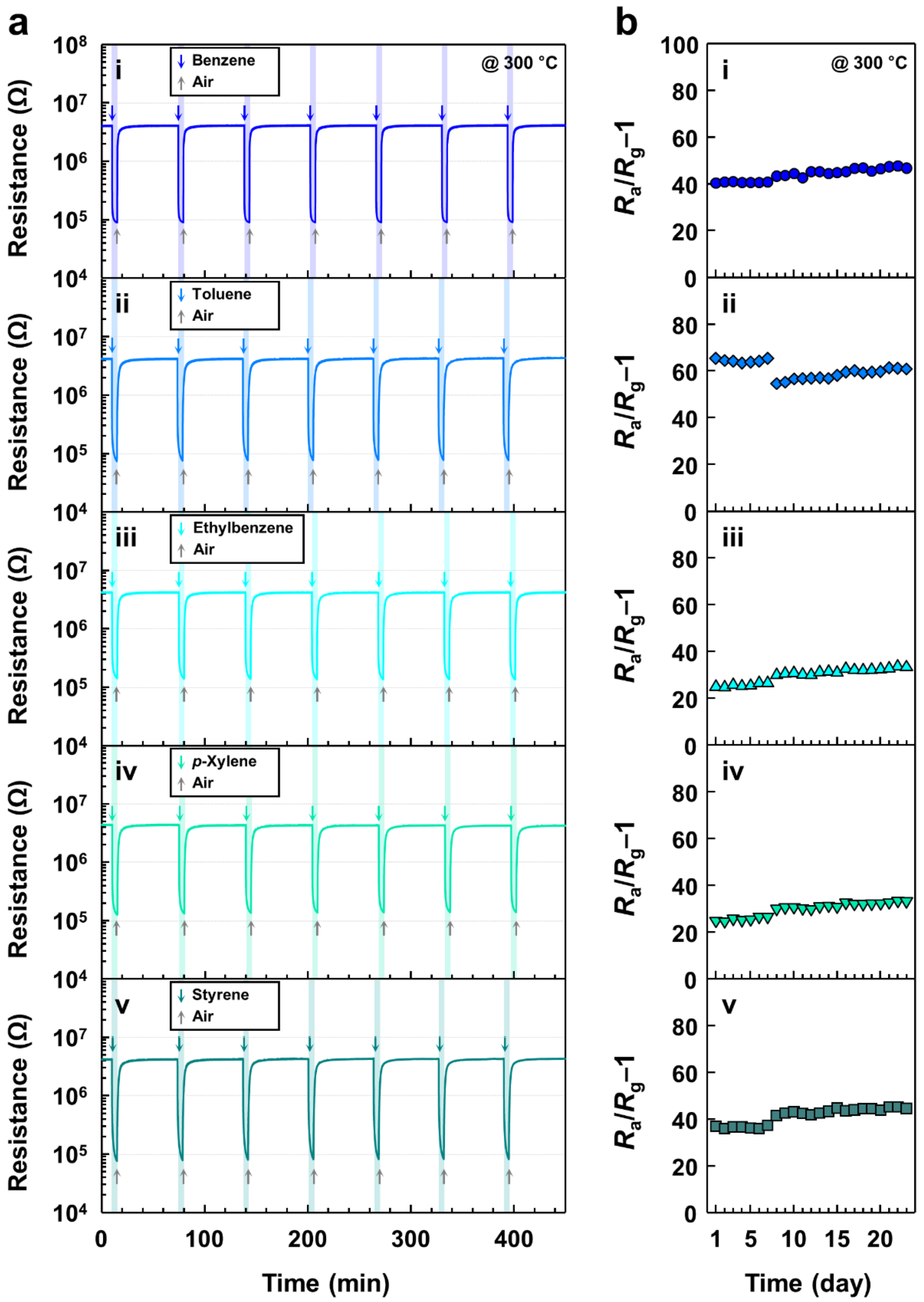

Supplementary Fig. 14 | a–b Seven repeated sensing transients (**a**) and long-term stabilities (**b**) of 0.4CeO_2/Rh–SnO_2 sensor (aromatic BTEXS gas concentrations: 5 ppm; temperature range: 300 °C).

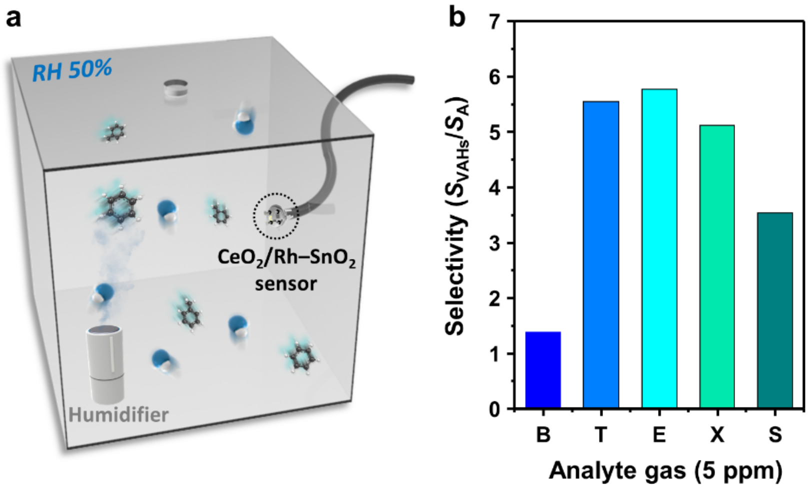

Supplementary Fig. 15 | a Schematic of sensor measurement in simulated conditoin (relative humidity: 50% at 18 °C). **b** Gas-sensing charactersitics (VAH selectivitgy over ethanol) of $0.4CeO_2/Rh–SnO_2$ sensor under humidity condition (B: benzene selectivity over ethanol, T: toluene selectivity over ethanol, E: ethylbenzene selectivity over ethanol, X: *p*-xylene selectivity over ethanol, S: styrene selectivity over ethanol). An acrylic chamber with a fixed volume (inner volume: 50 cm x 50 cm x 50 cm) was used for measuring gas sensing characteristics. The humidity in the chamber was controlled by miniaturized humidifier.

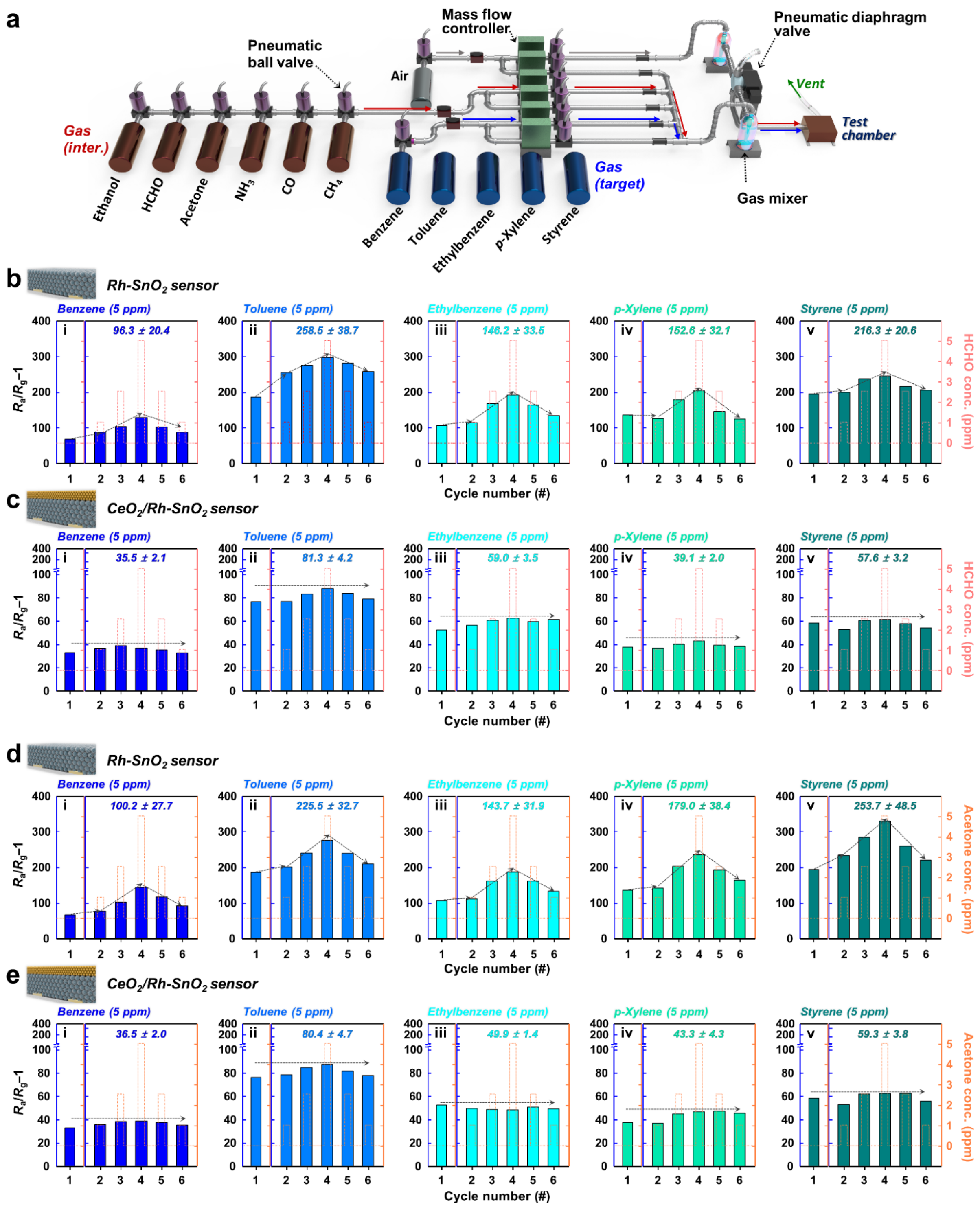

Supplementary Fig. 16 | a Schematic of gas-mixing system. **b–c** Gas responses of Rh–SnO_2 (**b**) and 0.4CeO_2/Rh–SnO_2 (**c**) sensors to gas mixtures comprising 5 ppm of volatile aromatic hydrocarbons ((i) benzene; (ii) toluene; (iii) ethylbenzene; (iv) *p*-xylene; or (v) styrene) and 0–5 ppm HCHO at 300 °C. **d–e** Gas responses of Rh–SnO_2 (**d**) and 0.4CeO_2/Rh–SnO_2 (**e**) sensors to gas mixtures comprising 5 ppm of volatile aromatic hydrocarbons ((i) benzene; (ii) toluene; (iii) ethylbenzene; (iv) *p*-xylene; or (v) styrene) and 0–5 ppm acetone at 300 °C.

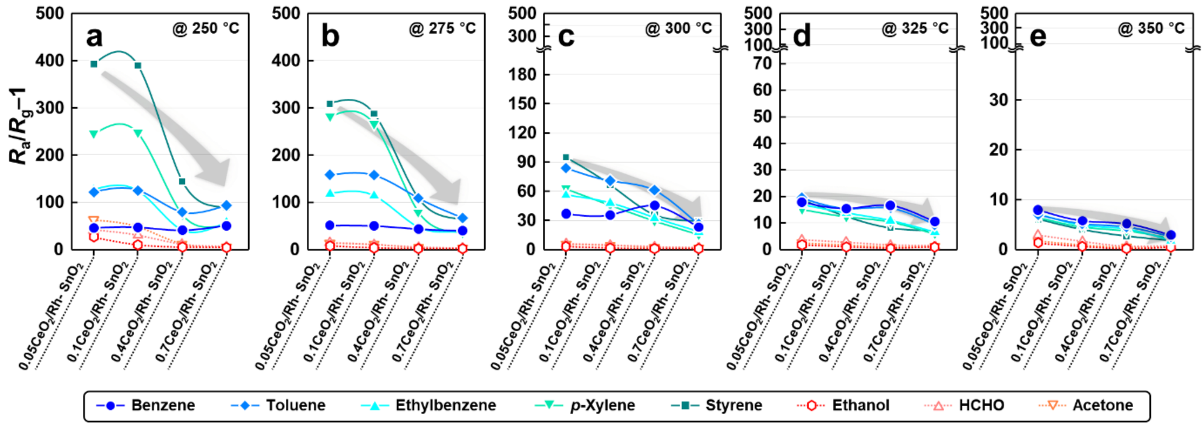

Supplementary Fig. 17 | a–e Gas responses to aromatic compounds (concentration: 5 ppm) plotted as functions of CeO_2 overlayer thickness for Rh–SnO_2, 0.05CeO_2/Rh–SnO_2, 0.1CeO_2/Rh–SnO_2, 0.4CeO_2/Rh–SnO_2, and 0.7CeO_2/Rh–SnO_2 sensors in range 250–350 °C (250 °C (**a**); 275 °C (**b**); 300 °C (**c**); 325 °C (**d**); and 350 °C (**e**)).

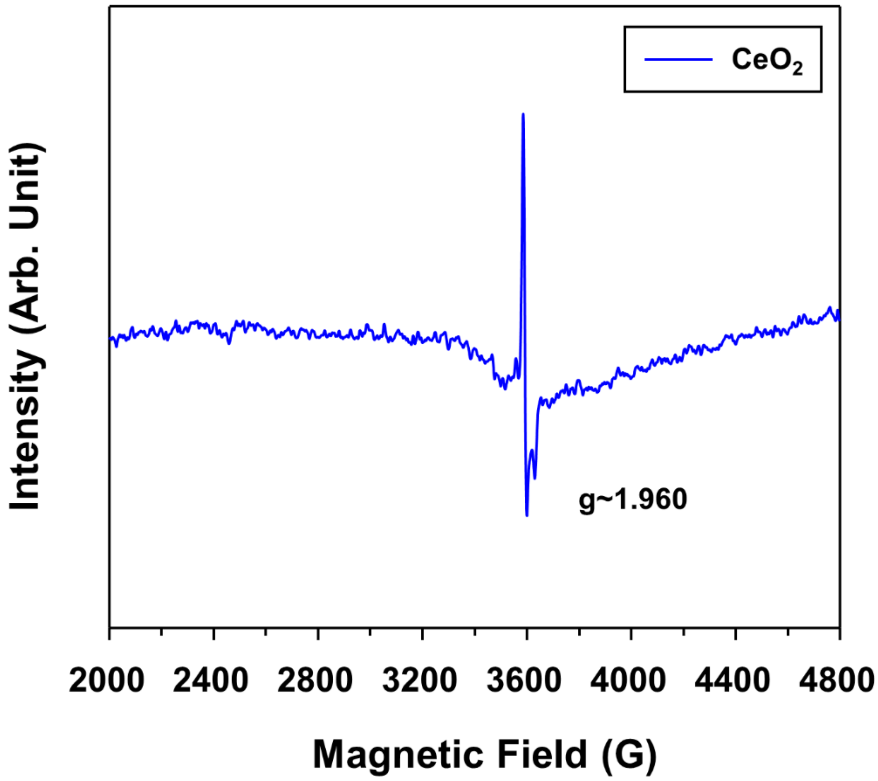

Supplementary Fig. 18 | EPR spectrum of CeO_2.

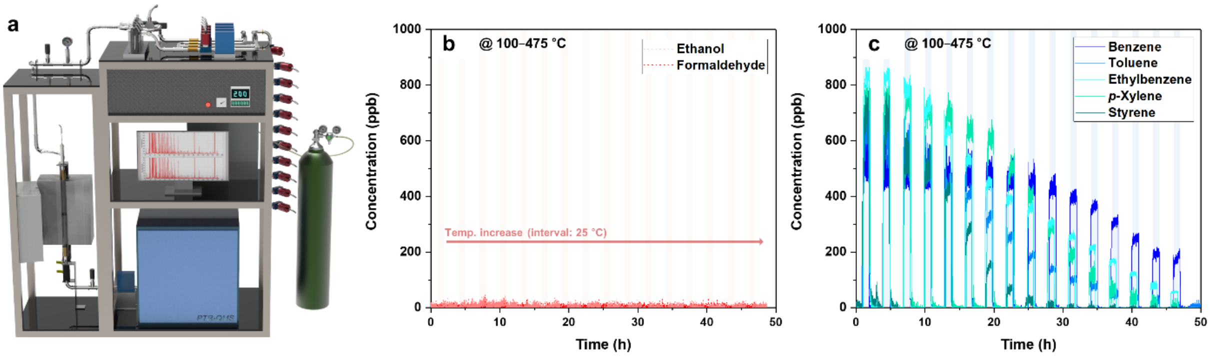

Supplementary Fig. 19 | a Schematic of PTR–QMS equipment setup. **b**–**c** Outlet gas concentrations of interfering gases (ethanol and HCHO) (**b**) and aromatic compounds (benzene, toluene, ethylbenzene, *p*-xylene, and styrene) (**c**) in the presence of CeO_2 (temperature range: 100–475 °C).

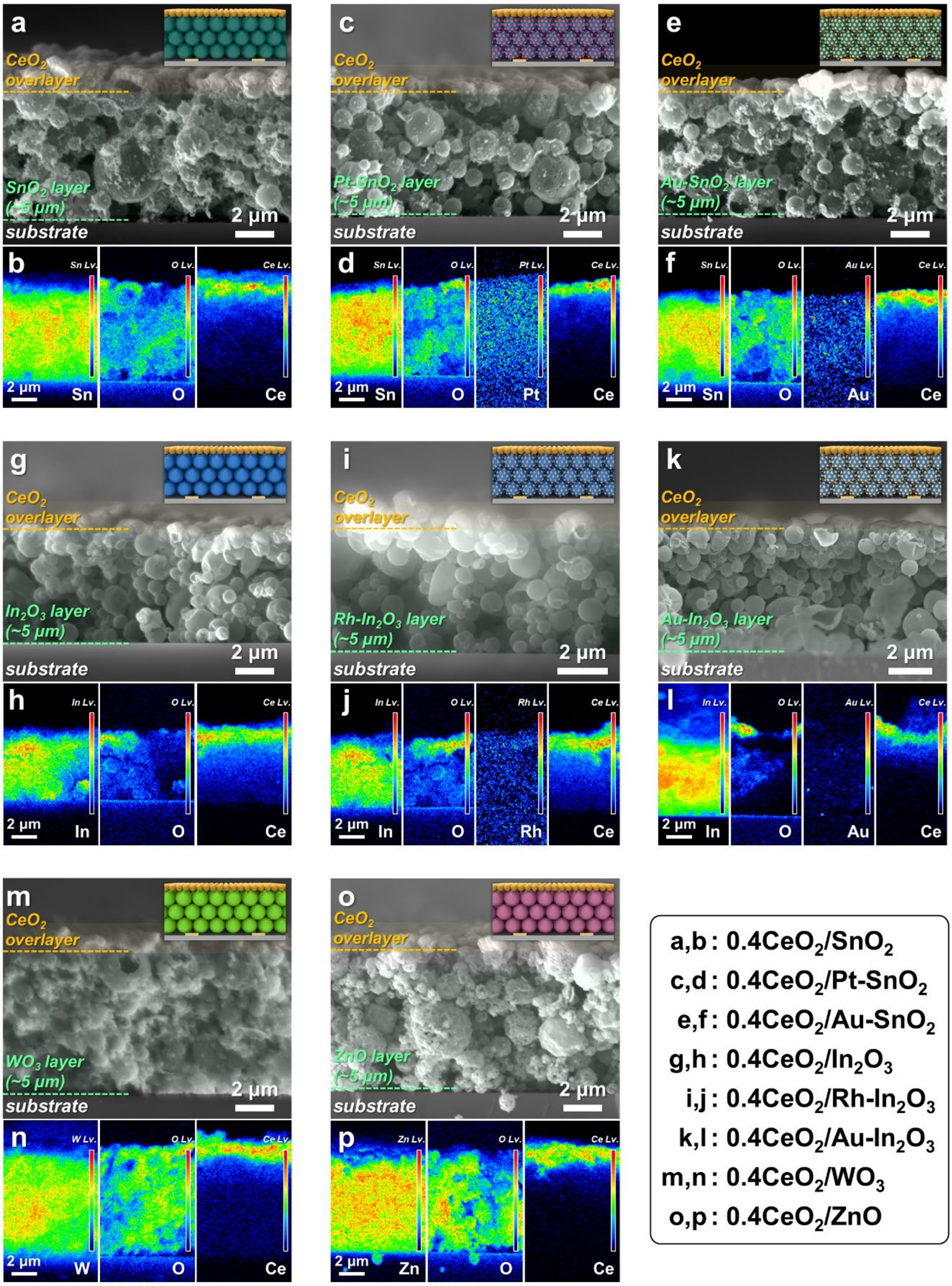

Supplementary Fig. 20 | a–p Cross-sectional SEM and EPMA elemental mapping images of $0.4CeO_2/SnO_2$ (**a** and **b**), $0.4CeO_2/Pt–SnO_2$ (**c** and **d**), $0.4CeO_2/Au–SnO_2$ (**e** and **f**), $0.4CeO_2/In_2O_3$ (**g** and **h**), $0.4CeO_2/Rh–In_2O_3$ (**i** and **j**), $0.4CeO_2/Au–In_2O_3$ (**k** and **l**), $0.4CeO_2/WO_3$ (**m** and **n**), and $0.4CeO_2/ZnO$ films (**o** and **p**).

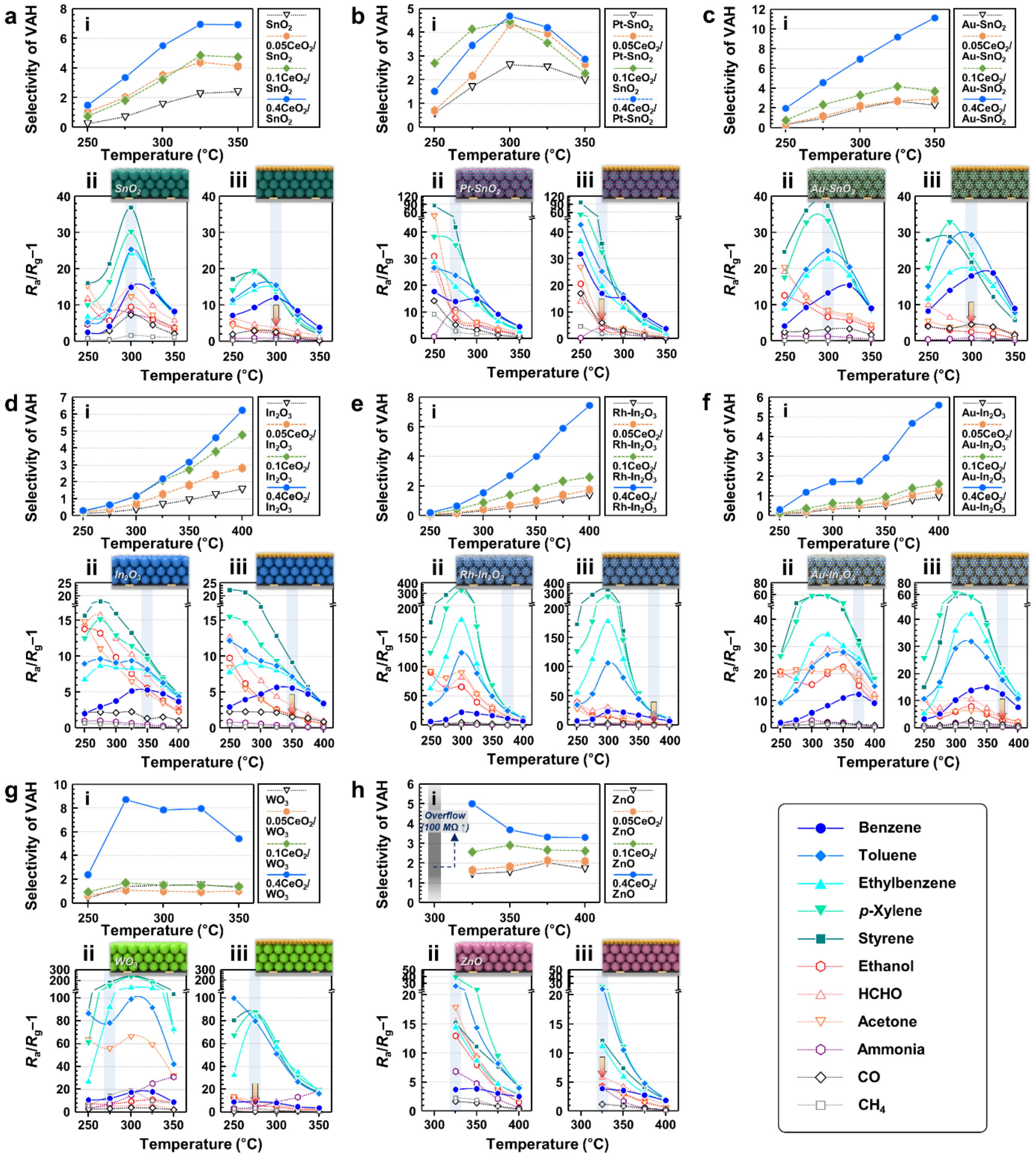

Supplementary Fig. 21 | a–h (i) VAH selectivity (S_{VAH}/S_A) and gas-sensing properties of diverse (ii) single-layer sensors and (iii) CeO_2-coated bilayer sensors (sensing layer material: SnO_2 (**a**), Pt–SnO_2 (**b**), Au–SnO_2 (**c**), In_2O_3 (**d**), Rh–In_2O_3 (**e**), Au–In_2O_3 (**f**), WO_3 (**g**), and ZnO (**h**); CeO_2 overlayer thickness: 0.05–0.4 μm thick; analyte gas concentration: 5 ppm; temperature range: 250–400 °C).

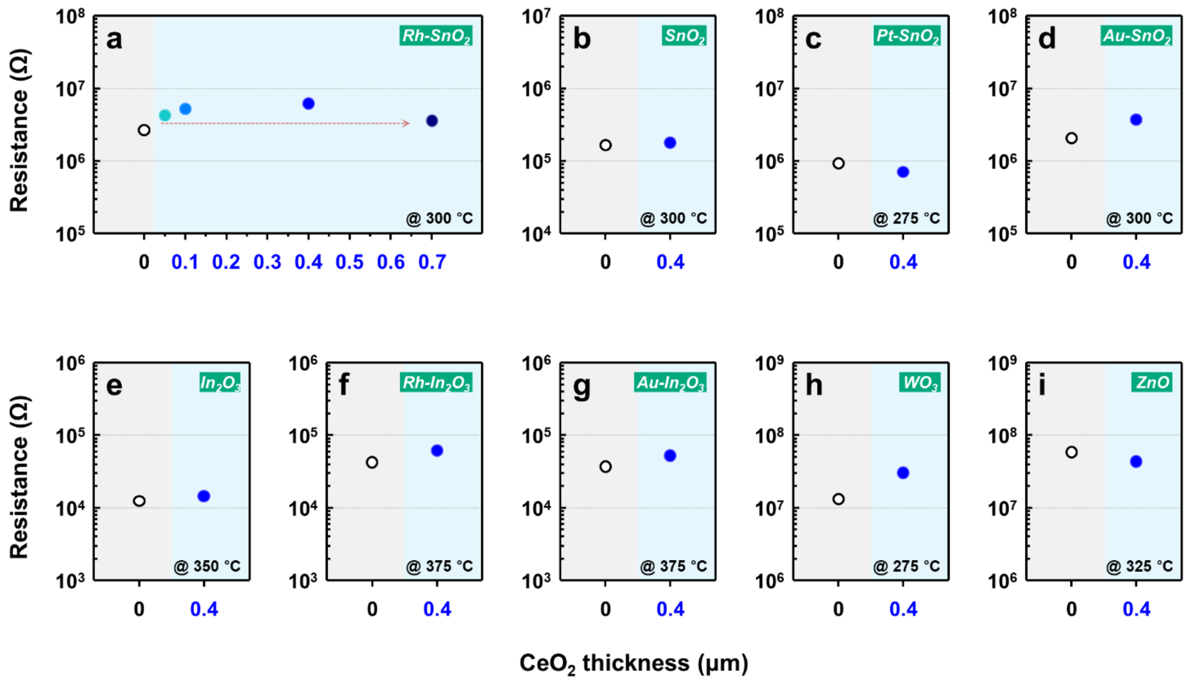

Supplementary Fig. 22 | a–i Sensor resistances (R_a) of xCeO$_2$/Rh–SnO$_2$ [x = 0–0.7-μm-thick] (**a**), xCeO$_2$/SnO$_2$ [x = 0- or 0.4-μm-thick] (**b**), xCeO$_2$/Pt–SnO$_2$ [x = 0- or 0.4-μm-thick] (**c**), xCeO$_2$/Au–SnO$_2$ [x = 0- or 0.4-μm-thick] (**d**), xCeO$_2$/In$_2$O$_3$ [x = 0- or 0.4-μm-thick] (**e**), xCeO$_2$/Rh–In$_2$O$_3$ [x = 0- or 0.4-μm-thick] (**f**), xCeO$_2$/Au–In$_2$O$_3$ [x = 0- or 0.4-μm-thick] (**g**), xCeO$_2$/WO$_3$ [x = 0- or 0.4-μm-thick] (**h**), and xCeO$_2$/ZnO [x = 0- or 0.4-μm-thick] (**i**) sensors in air.

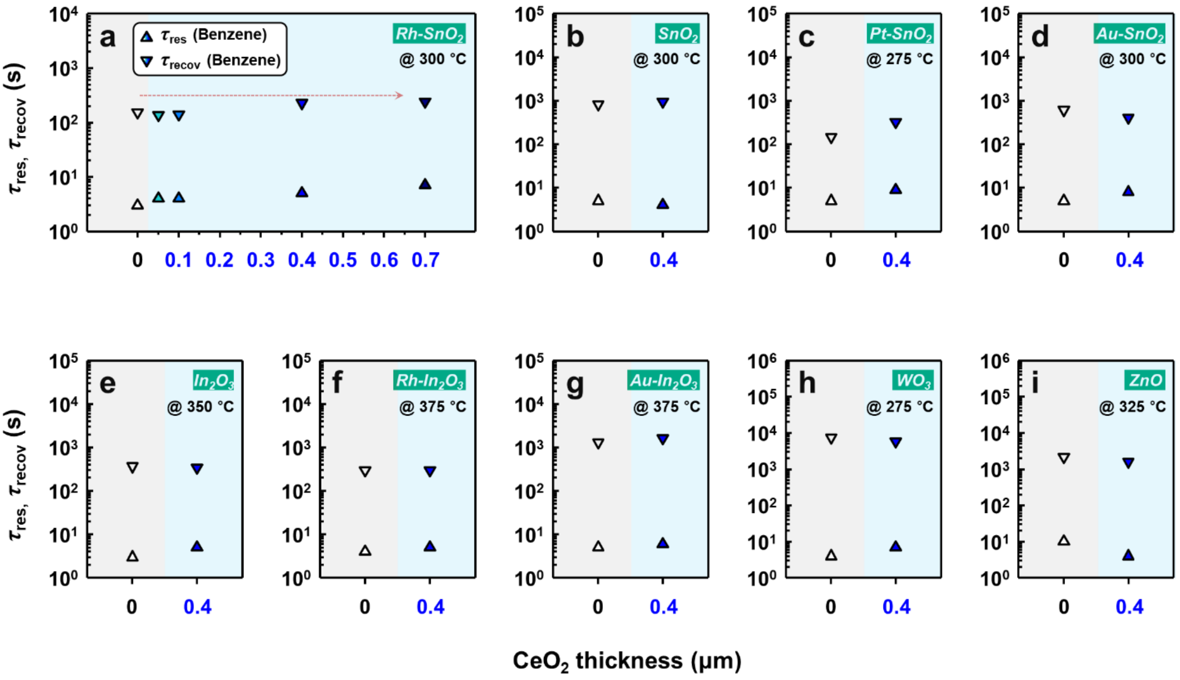

Supplementary Fig. 23 | a–i Ninety percent (90%) response and recovery times (τ_{res} and τ_{recov}, respectively) of *x*CeO_2/Rh–SnO_2 [*x* = 0–0.7-μm-thick] (**a**), *x*CeO_2/SnO_2 [*x* = 0- or 0.4-μm-thick] (**b**), *x*CeO_2/Pt–SnO_2 [*x* = 0- or 0.4-μm-thick] (**c**), *x*CeO_2/Au–SnO_2 [*x* = 0- or 0.4-μm-thick] (**d**), *x*CeO_2/In_2O_3 [*x* = 0- or 0.4-μm-thick] (**e**), *x*CeO_2/Rh–In_2O_3 [*x* = 0- or 0.4-μm-thick] (**f**), *x*CeO_2/Au–In_2O_3 [*x* = 0- or 0.4-μm-thick] (**g**), *x*CeO_2/WO_3 [*x* = 0- or 0.4-μm-thick] (**h**), and *x*CeO_2/ZnO [*x* = 0- or 0.4-μm-thick] (**i**) sensors.

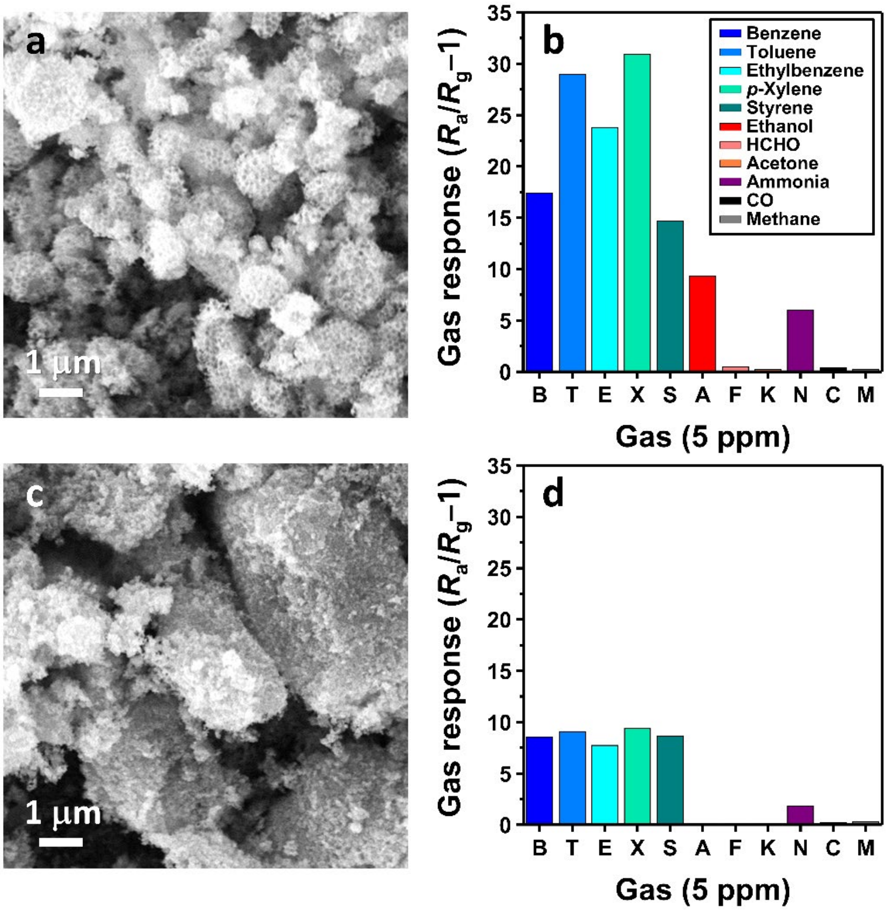

Supplementary Fig. 24 | a–b SEM image of the hierarchically porous Rh–SnO_2 (**a**), and gas sensing characteristics of CeO_2/hierarchically porous Rh–SnO_2 sensor at 300 °C (**b**). **c–d** SEM image of the nanoparticle Rh–SnO_2 (**c**), and gas sensing characteristics of CeO_2/nanoparticle Rh-SnO_2 sensor at 400 °C (**d**) (CeO_2 overlayer thickness: 0.4 μm thick; analyte gas concentration: 5 ppm).

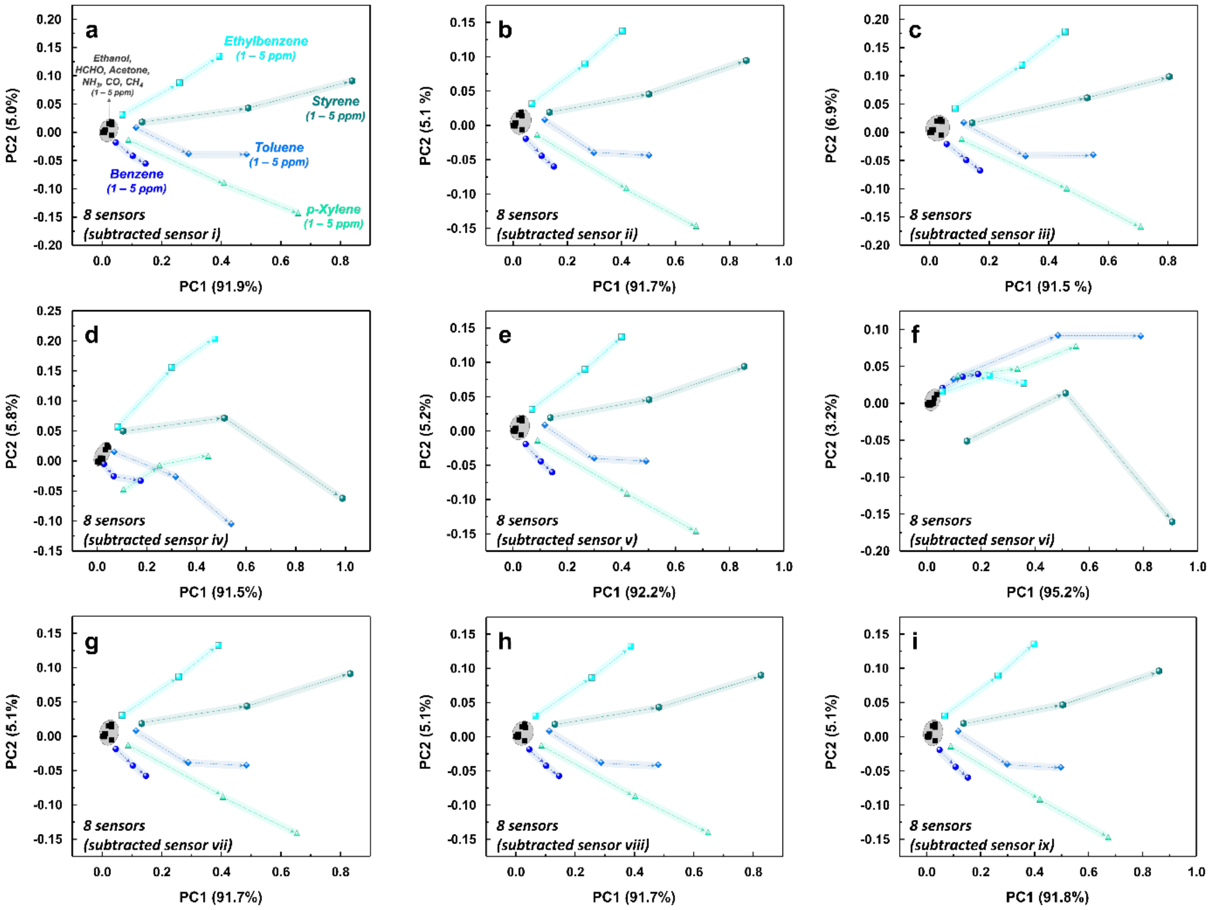

Supplementary Fig. 25 | a–i PCA results constructed by gas response patterns of the 8 sensors subtracted from the 9 sensors array in Fig. 9; Subtracted i (**a**), subtracted ii (**b**), subtracted iii (**c**), subtracted iv (**d**), subtracted v (**e**), subtracted vi (**f**), subtracted vii (**g**), subtracted viii (**h**), and subtracted ix (**i**).

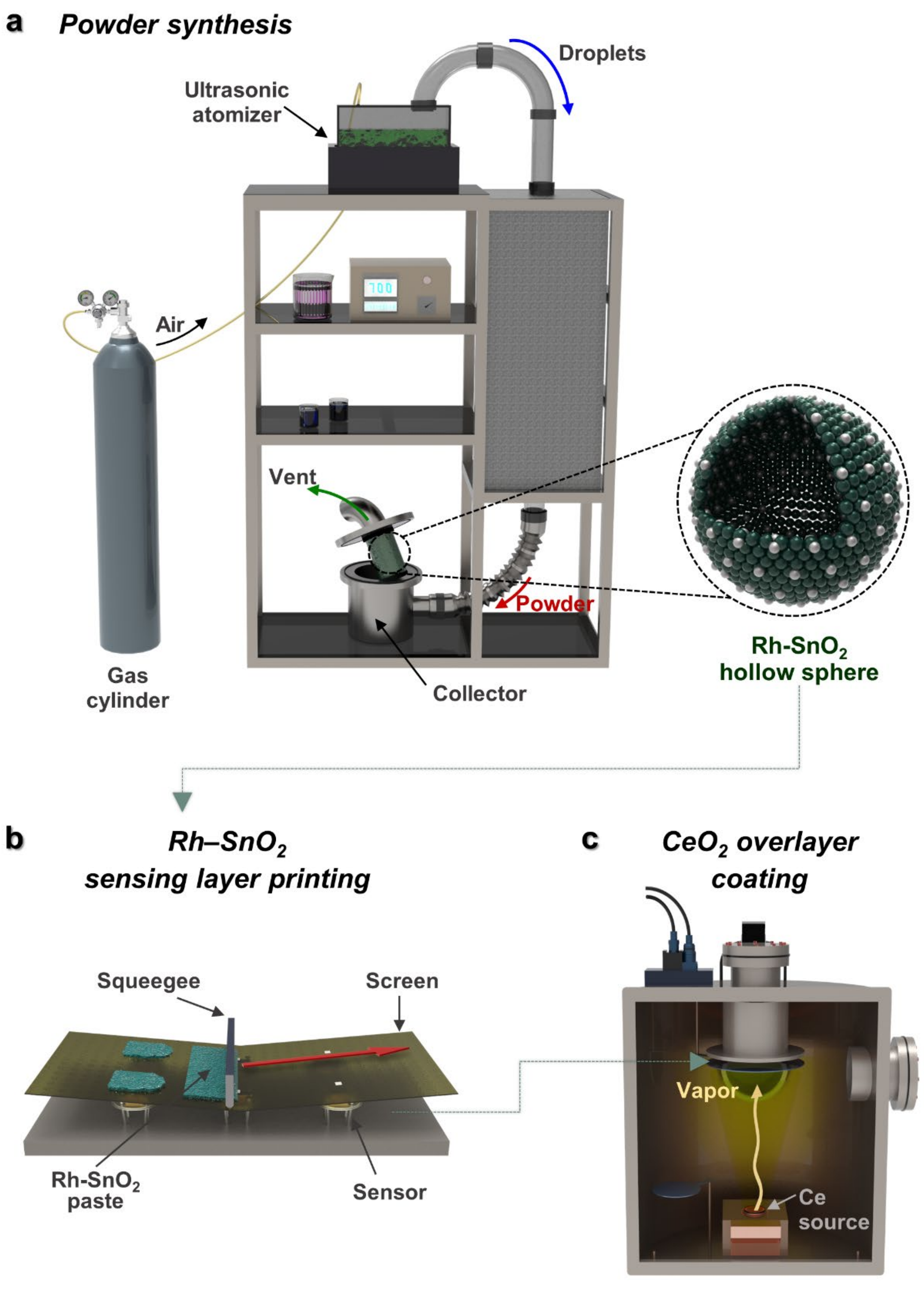

Supplementary Fig. 26 | a–c Schematic of overall experimental procedure (spray pyrolysis (**a**); screen printing (**b**); and e-beam evaporation (**c**)).

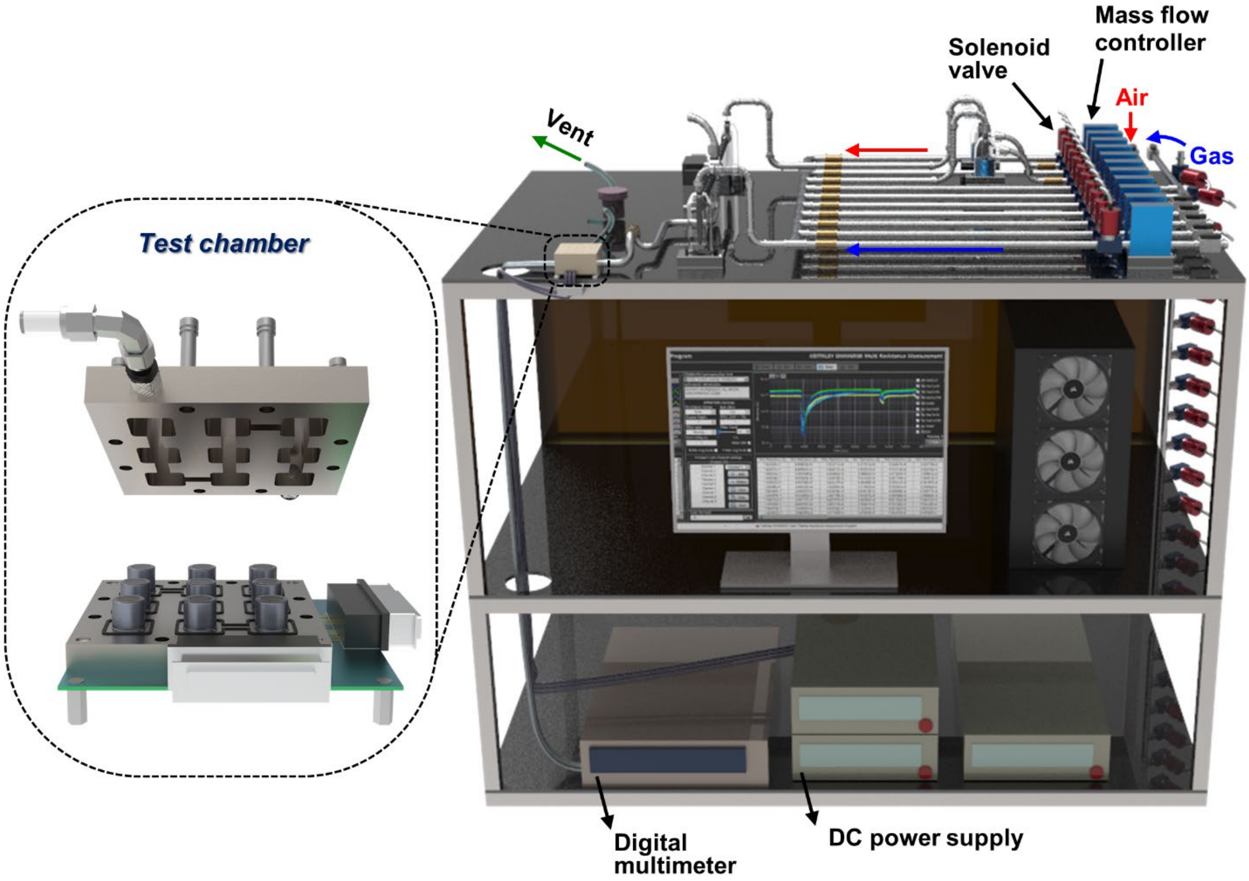

Supplementary Fig. 27 | Schematic of gas-sensor measurement system.

Supplementary Table 1 | Properties of various materials used for sensing gaseous volatile aromatic hydrocarbons, as reported in literature and obtained in this study[37,42–45,S9–S23].

**Material**	**Gas**	**Conc. [ppm]**	**Sensitivity [δS/δC]**	**Response [$R_aR_g^{-1}-1$, $R_gR_a^{-1}-1$, $I_aI_g^{-1}-1$]**	**VAH Selectivity**	**Sensor temp. [°C]**	**R_a [MΩ]**	**Detection limit [ppm]**	**Ref.**
CuO	Benzene	240	1.27	0.07	~1.0 to ~5.0	160	-	-	[S9]
nanoparticles	Toluene		0.43	0.35			-	-	
	Ethylbenzene		0.69	0.16			-	-	
	Xylene		0.51	0.19			-	-	
	Styrene		-	-	-	-	-	-	
CuO/SnO_2	Benzene	50	0.42	~5.2	~1.3 to ~2.2	280	-	10	[S10]
composites	Toluene		0.39	~7.0				-	
	Ethylbenzene		0.41	~7.1				-	
	Xylene		0.37	~9.0				-	
	Styrene		-	-	-	-	-	-	
SnO_2/V_2O_5	Benzene	50	0.37	~4.3	~2.0	270	-	-	[S11]
composites	Toluene		0.35	~4.5			-	-	
	Ethylbenzene		0.36	~5.0			-	-	
	Xylene		0.35	~5.4			-	-	
	Styrene		-	-	-	-	-	-	
Hexagonal	Benzene	50	0.60	11.3	~3.0 to	320	-	-	[S12]
WO_3	Toluene		0.57	26.3	~14.0		-	-	
nanosheets	Ethylbenzene		0.40	42.2			-	-	
	Xylene		0.49	35.3			-	-	
	Styrene	-	-	-	-	-	-	-	
Triangular-	Benzene	50	-	9.3	~9.0 to	200	15.0	10	[S13]
CeO_2-	Toluene		0.87	19.3	~19.0			-	
nanoflake-	Ethylbenzene		-	18.9				-	
coated ZnO	Xylene		-	18.7				-	
sensor	Styrene		-	-	-	-	-	-	
Cr-doped	Benzene	5	-	3.1	1.7	275	0.005	-	[S14]
Co_3O_4	Toluene		0.63	10.5	5.7			-	
nanorods	Ethylbenzene		-	-	-	-	-	-	
	Xylene		0.48	11.1	6.0			-	
	Styrene		-	-	-	-	-	-	
Pd-decorated	Benzene	1	-	25.5	~4.7	300	~0.8	-	[S15]
SnO_2	Toluene		-	2.3	~0.4			-	
nanowires	Ethylbenzene		-	-	-	-	-	-	
	Xylene		-	-	-	-	-	-	
	Styrene		-	-	-	-	-	-	

**Material**	**Gas**	**Conc. [ppm]**	**Sensitivity [δS/δC]**	**Response [$R_aR_g^{-1}$ –1, $R_gR_a^{-1}$ –1, $I_aI_g^{-1}$ –1]**	**VAH Selectivity**	**Sensor temp. [°C]**	**R_a [MΩ]**	**Detection limit [ppm]**	**Ref.**
Boron-nitride-	Benzene	100	0.19	~1.8	-	370	-	-	[S16]
quantum-dot-	Toluene		0.3	~2.0	-		-	-	
decorated ZnO	Ethylbenzene		0.19	~0.9	-		-	-	
nanoplates	Xylene		0.37	~2.9	-		-	-	
	Styrene		-	-	-	-	-	-	
Sea-urchin-like	Benzene	100	0.51	5	-	150	-	0.421	[S17]
TiO_2	Toluene		0.66	12.6	-		-	0.391	
hierarchical	Ethylbenzene		0.58	6.0	-		-	0.120	
nanostructures	Xylene		0.72	11.1	-		-	0.459	
	Styrene		-	-	-	-	-	-	
Co_3O_4 flowers	Benzene	50	0.33	26.2	-	200	-	-	[S18]
	Toluene		0.68	79.5	-	200	-	-	
	Ethylbenzene		-	-	-	-	-	-	
	Xylene		0.52	59	-	220	-	-	
	Styrene		-	-	-	-	-	-	
Au-decorated	Benzene	20	0.11	15.3	-	206	~130	10	[S19]
hierarchical	Toluene		0.48	45.4	-			-	
ZnO porous	Ethylbenzene		-	-	-	-	-	-	
rose-like	Xylene		0.64	75.4	-			-	
architectures	Styrene		-	-	-	-	-	-	
Cobalt–	Benzene	9	0.49	4.9	-	330	1950	5	[S20]
porphyrin-	Toluene		0.45	12.5	-			-	
functionalized	Ethylbenzene		-	-	-	-	-	-	
TiO_2	Xylene		0.35	13.2	-			-	
nanoparticles	Styrene		-	-	-	-	-	-	
α-Fe_2O_3	Benzene	0.1	-	0.42		280	~1200	3	[S21]
microrhombuses	Toluene		-	0.42					
	Ethylbenzene		-	0.42					
	Xylene		-	0.42					
	Styrene		-	-	-	-	-	-	
Au-decorated	Benzene	0.1	0.54	~0.00037	-	280	0.0006	0.0006	[S22]
oxygen-plasma-	Toluene		-	~0.00004	-			-	
treated	Ethylbenzene		-	-	-	-	-	-	
multiwalled	Xylene		-	~0.00001	-			-	
carbon nanotubes	Styrene		-	-	-	-	-	-	

Material	Gas	Conc. [ppm]	Sensitivity [δS/δC]	Response [$R_aR_g^{-1}-1$, $R_gR_a^{-1}-1$, $I_aI_g^{-1}-1$]	VAH Selectivity	Sensor temp. [°C]	R_a [MΩ]	Detection limit [ppm]	Ref.
Pentiptycene polymer/single-walled carbon nanotube complexes	Benzene	100	0.91	~0.0018	-	RT	~0.02 to ~1	-	[S23]
	Toluene		-	~0.0015	-			-	
	Ethylbenzene		-	-	-	-	-	-	
	Xylene		-	~0.0007	-			-	
	Styrene		-	-	-	-	-	-	
Au-loaded hierarchical MoO_3 hollow spheres	Benzene	100	0.22	4.3	1.1	250	~840	5	[37]
	Toluene		0.69	16.5	4.1			0.1	
	Ethylbenzene		-	-	-	-	-	-	
	Xylene		1.05	21.1	5.3			0.5	
	Styrene		-	-	-	-	-	-	
Co_3O_4/Pd–SnO_2 sensor	Benzene	5	0.71	87.0	6.0	375	2.9	-	[42]
	Toluene		-	28.5	2.0			-	
	Ethylbenzene		-	-	-	-	-	-	
	Xylene		-	16.7	1.2			-	
	Styrene		-	-	-	-	-		
TiO_2/Co_3O_4 sensor	Benzene	5	-	1.5	1.6	250	0.0014	-	[43]
	Toluene		-	10.7	7.5				
	Ethylbenzene		-	-	-	-	-	-	
	Xylene		-	13.5	9.3				
	Styrene		-	-	-	-	-	-	
SnO_2/Co_3O_4 sensor	Benzene	5	-	1.7	1.3	250	0.0018	-	[44]
	Toluene		-	15.2	8.0				
	Ethylbenzene		-	-	-	-	-	-	
	Xylene		-	27.8	14.2				
	Styrene		-	-	-	-	-	-	
Rh–TiO_2/SnO_2 sensor	Benzene	5	1.23	33.4	14.0	325	~5.0	-	[45]
	Toluene		-	8.4	3.5			-	
	Ethylbenzene		-	-	-	-	-	-	
	Xylene		-	4.0	1.7			-	
	Styrene	-	-	-	-	-	-	-	
Au/SnO_2 sensor	Benzene	5	-	23.1	1.8	350	3.0	-	[46]
	Toluene		-	56.2	4.3				
	Ethylbenzene		-	-	-	-	-	-	
	Xylene		0.92	61.4	4.6				
	Styrene		-	-	-	-	-	-	

**Material**	**Gas**	**Conc. [ppm]**	**Sensitivity [δS/δC]**	**Response [$R_aR_g^{-1}$ –1, $R_gR_a^{-1}$ –1, $I_aI_g^{-1}$ –1]**	**VAH Selectivity**	**Sensor temp. [°C]**	**R_a [MΩ]**	**Detection limit [ppm]**	**Ref.**
$0.4CeO_2/WO_3$ sensor	Benzene	5	0.81	9.1	1.4	275	~32.3	-	This study
	Toluene		0.99	79.7	12.3			-	
	Ethylbenzene		0.69	87.5	12.8			-	
	Xylene		1.13	83.0	13.5			-	
	Styrene		1.01	87.5	13.5			-	
$0.4CeO_2$/Rh–In_2O_3 sensor	Benzene	5	0.48	23.8	1.5	300	~0.062	-	This study
	Toluene		0.61	106.8	6.9			-	
	Ethylbenzene		1.00	178.5	11.6			-	
	Xylene		1.00	269.3	17.5			-	
	Styrene		1.24	338.5	22.0			-	
$0.05CeO_2$/Rh–SnO_2 sensor	Benzene	5	0.67	51.2	6.9	275	~4.2	-	This study
	Toluene		0.90	158.2	21.4			-	
	Ethylbenzene		1.12	118.5	16.0			-	
	Xylene		1.25	281.5	38.1			-	
	Styrene		1.27	308.8	41.8			-	
$0.1CeO_2$/Rh–SnO_2 sensor	Benzene	5	0.97	50.2	14.7	275	~5.2	-	This study
	Toluene		1.24	157.8	46.2			-	
	Ethylbenzene		1.42	114.0	33.4			-	
	Xylene		1.61	265.5	77.7			-	
	Styrene		1.01	287.3	84.0			-	
$0.4CeO_2$/Rh–SnO_2 sensor	Benzene	5	0.86	44.7	44.7	300	~6.1	0.081	This study
	Toluene		0.92	60.5	60.5			0.089	
	Ethylbenzene		0.82	32.3	32.6			0.132	
	Xylene		0.75	28.8	28.8			0.107	
	Styrene		0.81	35.0	35.0			0.100	
$0.7CeO_2$/Rh–SnO_2 sensor	Benzene	5	0.77	39.7	23.2	275	~3.6	-	This study
	Toluene		1.21	66.8	39.0			-	
	Ethylbenzene		1.23	38.5	22.5			-	
	Xylene		1.41	34.4	20.1			-	
	Styrene		1.81	63.4	37.0			-	

References

S1. Jeong, Y.J. et al. Chitosan-templated Pt nanocatalyst loaded mesoporous SnO_2 nanofibers: a superior chemiresistor toward acetone molecules. *Nanoscale* **10**, 13713-13721 (2018).

S2. Park, S.-W., Jeong, S.-Y., Yoon, J.-W. & Lee, J.-H. General Strategy for Designing Highly Selective Gas-Sensing Nanoreactors: Morphological Control of SnO_2 Hollow Spheres and Configurational Tuning of Au Catalysts. *ACS Appl. Mater. Interfaces* **12**, 51607-51615 (2020).

S3. Wolkoff, P. & Nielsen, G.D. Organic compounds in indoor air—their relevance for perceived indoor air quality?. *Atmospheric Environ.* **35**, 4407-4417 (2001).

S4. Obee, T.N. & Brown, R.T. TiO_2 photocatalysis for indoor air applications: effects of humidity and trace contaminant levels on the oxidation rates of formaldehyde, toluene, and 1,3-butadiene. *Environ. Sci. Technol.* **29**, 1223-1231 (1995).

S5. Yuan, Z. et al. Trace-Level, Multi-Gas Detection for Food Quality Assessment Based on Decorated Silicon Transistor Arrays. *Adv. Mater.* **32**, 1908385 (2020).

S6. Jay, J.M. Mechanism and detection of microbial spoilage in meats at low temperatures: A status report. *J. Milk Food Technol.* **35**, 467-471 (1972).

S7. Buyukkaya, E. Effects of biodiesel on a DI diesel engine performance, emission and combustion characteristics. *Fuel* **89**, 3099-3105 (2010).

S8. Schwietzke, S. et al. Upward revision of global fossil fuel methane emissions based on isotope database. *Nature* **538**, 88-91 (2016).

S9. Thangamani, J.G. & Pasha, S.K.K. Hydrothermal synthesis of copper (II) oxide-nanoparticles with highly enhanced BTEX gas sensing performance using chemiresistive sensor. *Chemosphere* **277**, 130237 (2021).

S10. Ren, F. et al. Enhanced BTEX gas-sensing performance of CuO/SnO_2 composite. *Sens. Actuators, B* **223**, 914-920 (2016).

S11. Zhang, F., Wang, X., Dong, J., Qin, N. & Xu, J. Selective BTEX sensor based on a SnO_2/V_2O_5 composite. *Sens. Actuators, B* **186**, 126-131 (2013).

S12. Zhang, D. et al. Highly sensitive BTEX sensors based on hexagonal WO_3 nanosheets. *Sens. Actuators, B* **293**, 23-30 (2019).

S13. Wang, D. et al. The catalytic-induced sensing effect of triangular CeO_2 nanoflakes for enhanced BTEX vapor detection with conventional ZnO gas sensors. *J. Mater. Chem. A* **8**, 11188-11194 (2020).

S14. Jeong, H.-M., Kim, H.-J., Rai, P., Yoon, J.-W. & Lee, J.-H. Cr-doped Co_3O_4 nanorods as chemiresistor for ultraselective monitoring of methyl benzene. *Sens. Actuators, B* **201**, 482-489 (2014).

S15. Kim, J.-H., Wu, P., Kim, H.W. & Kim, S.S. Highly Selective Sensing of CO, C_6H_6, and C_7H_8 Gases by Catalytic Functionalization with Metal Nanoparticles. *ACS Appl. Mater. Interfaces* **8**, 7173-7183 (2016).

S16. Choudhury, S.P. et al. BN quantum dots decorated ZnO nanoplates sensor for enhanced detection of BTEX gases. *J. Alloys Compd.* **815**, 152376 (2020).

S17. Tshabalala, Z.P., Mokoena, T.P., Hillie, K.T., Swart, H.C. & Motaung, D.E. Improved BTEX gas sensing characteristics of thermally treated TiO_2 hierarchical spheres manifested by high-energy {001} crystal facets. *Sens. Actuators, B* **338**, 129774 (2021).

S18. Cao, J. et al. Porous nanosheets assembled Co_3O_4 hierarchical architectures for enhanced BTX (Benzene, Toluene and Xylene) gas detection. *Sens. Actuators, B* **315**, 128120 (2020).

S19. Shen, Z. et al. The significant improvement for BTX (benzene, toluene and xylene) sensing performance based on Au-decorated hierarchical ZnO porous rose-like architectures. *Sens. Actuators, B* **262**, 86-94 (2018).

S20. Kang, Y., Kim, K., Cho, B., Kwak, Y. & Kim, J. Highly Sensitive Detection of Benzene, Toluene, and Xylene Based on CoPP-Functionalized TiO_2 Nanoparticles with Low Power Consumption. *ACS Sens.* **5**, 754-763 (2020).

S21. Da Silva, L.F. et al. BTEX gas sensor based on hematite microrhombuses. *Sens. Actuators, B* **326**, 128817 (2021).

S22. Clément, P. et al. Deep Cavitand Self-Assembled on Au NPs-MWCNT as Highly Sensitive Benzene Sensing Interface. *Adv. Funct. Mater.* **25**, 4011-4020 (2015).

S23. Luo, S.-X.L., Lin, C.-J., Ku, K.H., Yoshinaga, K. & Swager, T.M. Pentiptycene Polymer/Single-Walled Carbon Nanotube Complexes: Applications in Benzene, Toluene, and o-Xylene Detection. *ACS Nano* **14**, 7297-7307 (2020).
